# Single-site binding of pyrene to poly(ester-imide)s incorporating long spacer-units: prediction of NMR resonance-patterns from a fractal model†

**DOI:** 10.1039/d0sc03730c

**Published:** 2020-10-09

**Authors:** Marcus Knappert, Tianqi Jin, Scott D. Midgley, Guanglu Wu, Oren A. Scherman, Ricardo Grau-Crespo, Howard M. Colquhoun

**Affiliations:** Department of Chemistry, University of Reading Whiteknights Reading RG6 6AD UK h.m.colquhoun@rdg.ac.uk; Melville Laboratory for Polymer Synthesis, Department of Chemistry, University of Cambridge Lensfield Road Cambridge CB2 1EW UK

## Abstract

Co-polycondensation of the diimide-based diols *N*,*N*′-bis(2-hydroxyethyl)hexafluoroisopropylidene-diphthalimide, (HFDI), and *N*,*N*′-bis(2-hydroxy-ethyl)naphthalene-1,4,5,8-tetracarboxylic-diimide, (NDI), with aliphatic diacyl chlorides ClOC(CH_2_)_*x*_COCl (*x* = 5 to 8) affords linear copoly(ester-imide)s. Such copolymers interact with pyrene *via* supramolecular binding of the polycyclic aromatic at NDI residues. This interaction results in upfield complexation shifts and sequence-related splittings of the NDI ^1^H NMR resonances, but gives a very different final resonance-pattern from the copolymer where *x* = 2. Computational modelling of the polymer with *x* = 5 suggests that each pyrene molecule binds to just a single NDI residue rather than by intercalation between a pair of NDI's at a tight chain-fold, as was found for *x* = 2. The new single-site binding model enables the pattern of ^1^H NMR resonances for copolymers with longer spacers (*x* = 5 to 8) to be reproduced and assigned by simulation from sequence-specific shielding factors based on a type of fractal known as the last-fraction Cantor set. As this type of fractal also enables an understanding of pairwise binding systems, it evidently provides a general numerical framework for supramolecular sequence-analysis in binary copolymers.

## Abstract 2

Nine ^1^H NMR resonances assignable to specific copoly(ester-imide) sequences identified from a fractal model result from 1 : 1 supramolecular binding of pyrene to NDI residues.

## Graphical abstract

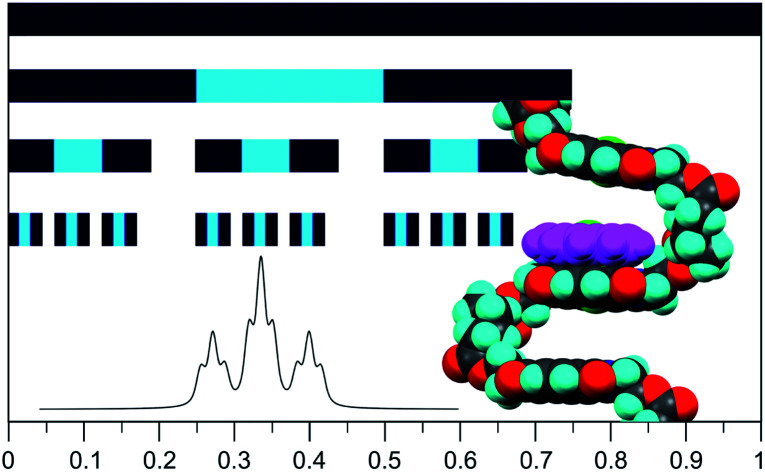

## 1. Introduction

The storage, copying and processing of information in biological systems is achieved, universally and with high precision at the molecular level, by a group of sequence-defined, high molecular-weight, linear copolymers (DNA and/or RNA, and proteins).^1–3^ In principle, however, any copolymer sequence can represent information, because even the simplest AB copolymer is the equivalent of a binary string.^4,5^ Indeed, some very significant progress in devising a synthetic “information chemistry” has been made in recent years, notably with the development of sequence-specific polymerisation (*i.e.* sequence-writing) methodologies and mass-spectrometric sequencing techniques,^6–8^ information-transfer protocols,^9–11^ and the use of small “reader-molecules” to recognise and report copolymer sequence-information.^12–15^

Using the latter technique, we recently showed that highly sequence-dependent ^1^H NMR complexation shifts are produced in the spectra of copolyimides based on 1,4,5,8-naphthalene tetracarboxylic diimide (NDI) on complexation of an aromatic “probe” molecule such as pyrene or perylene.^15,16^ This phenomenon results from cumulative ring-current shielding^17,18^ of the central residue in an NDI-centred sequence by probe-molecules binding through complementary π–π-stacking.^19–21^ Such shielding results not only from the probe-molecule binding directly at the central “observed” NDI residue but, additionally, from complexation to NDI residues at neighbouring (and next-neighbouring, and next–next-neighbouring *etc.*) positions, viewed in both directions from the centre of the sequence. As separate resonances corresponding to “bound” and “unbound” NDI residues are not observed at sub-stoichiometric levels of pyrene, the system is clearly operating in the fast-exchange regime.

Multiple NDI signals are seen at high pyrene concentrations, even under fast-exchange conditions, because each “observed” NDI residue is at the centre of a different but specific copolymer sequence. This sequence may in principle be of any length, although spectroscopic resolution generally limits the maximum “observable” length to a quintet or septet. This “surrounding sequence” defines the molecular and supramolecular environment of the central, observed NDI residue, because the distribution pattern of other NDI residues in the sequence determines the number and locations of all pyrene molecules binding to that sequence. Thus, NDI residues at the centres of different sequences are inherently distinct and give different complexation shifts in the presence of pyrene, regardless of exchange between bound and unbound states. It must be emphasised that, under the conditions of the experiment, there is no exchange of monomer residues between different sequences, and so an averaging of NDI resonances over all sequences is impossible.

The concept of a central “observed” residue in a given sequence is important, because it greatly simplifies sequence-analysis in high molecular weight copolymers. Even though a given copolymer sequence may contain many “observable” monomer residues (in the present context, NDI), each of these is also at the centre of its own sequence, overlapping with the original sequence but still, in NMR terms, representing a specific, intramolecular environment. Consequently, the central residue can be treated separately from other chemically equivalent residues in the same sequence. In NDI-based copolymers, ring-current shielding resulting from complexation of an aromatic molecule such as pyrene amplifies the differences between magnetic environments in a copolyimide chain and thus enables the assignment of specific NMR resonances to different comonomer sequences.^15^

**Fig. 1:**
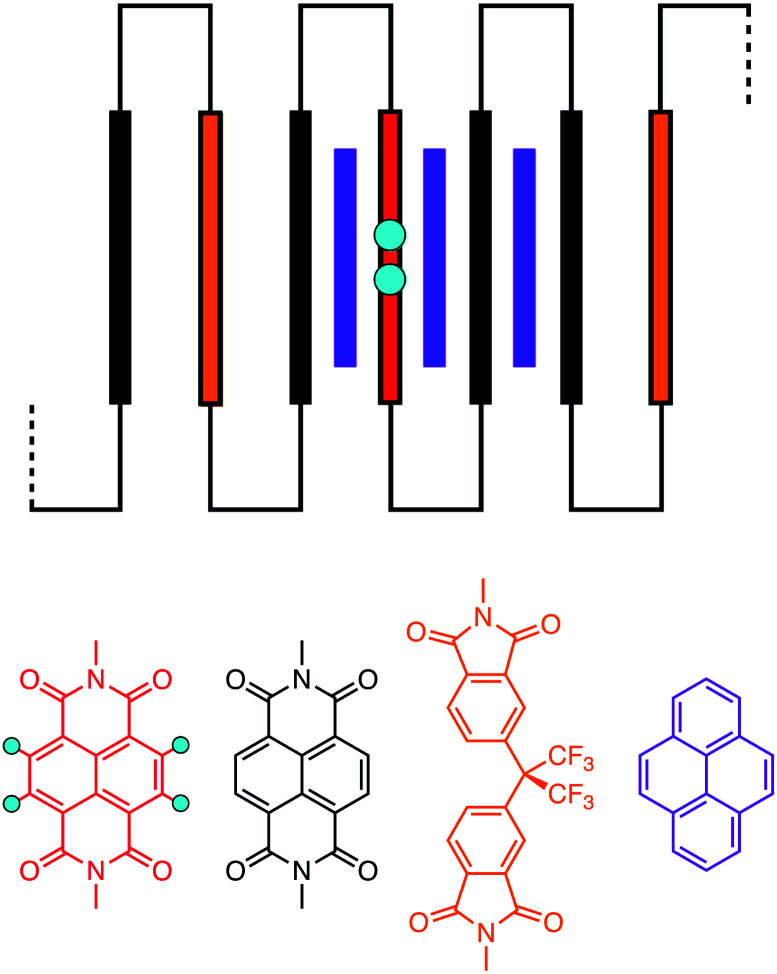
Schematic of pyrene binding to a chain-folding copoly(ester-imide) sequence.^16^ Key: purple = pyrene; blue = “observed” protons of the central NDI residue (red); black = other NDI residues; gold = non-binding (HFDI) residues. The aromatic diimide residues are linked by aliphatic diester units, [–CH_2_CH_2_OCO(CH_2_)_2_COOCH_2_CH_2_–], represented as thin black lines.

For copoly(ether-imide)s^15^ and copoly(ester-imide)s^16^ studied previously, it was found that tight chain-folding^22–24^ allowed the aromatic “reader-molecule” (generally pyrene) to bind strongly to the polymer chain by intercalation between adjacent NDI residues (Fig. 1).^25–27^ In this context, the chain-fold may be viewed as a “half-closed bis-diimide macrocycle”, related to the true NDI-based macrocycles reported by Sanders *et al.* as components of donor–acceptor catenanes with dialkoxynapthalenes as the donor groups.^28–30^ Somewhat related rotaxane complexes, comprising 4,4-bipyridinium macrocycles threaded onto a high-MW, chain-folding poly(dialkoxynaphthalene), have been reported by Hodge *et al.*,^31^ and discrete oligomer-analogues of these, showing complex dynamic behaviour in solution and unusual end-group disorder (leading to pseudo-polymeric crystal structures) in the solid state were more recently described by Stoddart and co-workers.^32,33^ However, the systems described in ref. 31–33 are homopolymers (or homo-oligomers) rather than copolymers, so there is no “sequence-information” present. Moreover, the molecule involved in binding to the polymer or oligomer is a bipyridinium-based macrocycle (“Blue Box”) that is threaded onto the chain. This macrocycle, unlike the probe molecule – pyrene – used in the present work, does not dissociate from the chain on the timescale of the NMR experiment so that fast bound/unbound exchange is not observed.

Remarkably, the patterns of ^1^H NMR resonances originating from NDI residues in random, binary copolyimide chains, in the presence of pyrene, exhibit a noticeable degree of self-similarity,^34,35^*i.e.* the spectra consist of multiple copies of themselves over a range of different length-scales.^15,16^ These resonance-patterns have been shown to reflect an underlying fractal distribution^34–37^ of ring-current shieldings generated by pyrene molecules binding to all the different sequences within which a “central” NDI residue is embedded.^15,16^

**Fig. 2:**
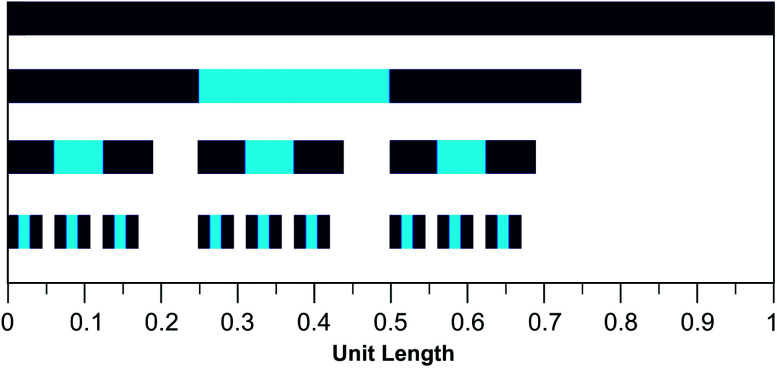
Graphical construction of the fourth-quarter Cantor set, showing the first three iterations. This construction involves dividing a line of unit length into four equal parts, discarding the fourth quarter, and repeating the procedure on the remaining three segments, through an infinity of iterations. In the limit, the construction converges to a maximum of 0.666…, but as shown above, the limit is approached after a relatively small number of iterations. Sets defined by a last-fraction construction of this type, though commonly described as “Cantor” sets, were actually discovered by Smith,^38^ and can be constructed using any number ≥3 (integral or non-integral) as the base.

The mathematical fractal underpinning the pattern of complexation shifts in such systems was identified^15^ as a “last fraction” Cantor set. Interestingly, this was the earliest class of fractal to be discovered (by Smith in 1875).^38–41^ An atomistic model for copolymer–pyrene complexation was developed in which ring-current shielding falls off, empirically, by a factor of four as pyrene binds to NDI pairs successively more distant from the central, “observed” NDI residue (Fig. 1).^15,16^ This “factor of four” in turn defined the set of all predicted complexation shifts, mathematically, as the fourth-quarter Cantor set. A complete mathematical description of this set is given in Section 3.3 and in the ESI† but, as with all one-dimensional Cantor sets, it can be visualised graphically by the iteration of an operation on a line. In this instance we divide a line of unit length into four equal parts, discard the fourth quarter, and repeat the procedure on the three remaining segments. The full set is obtained only after an infinity of iterations, but the group of “remaining segments” converges rapidly (in the absence of magnification) to a visually unchanging pattern. The first three iterations of the construction are shown in Fig. 2.

**Chart 1:**
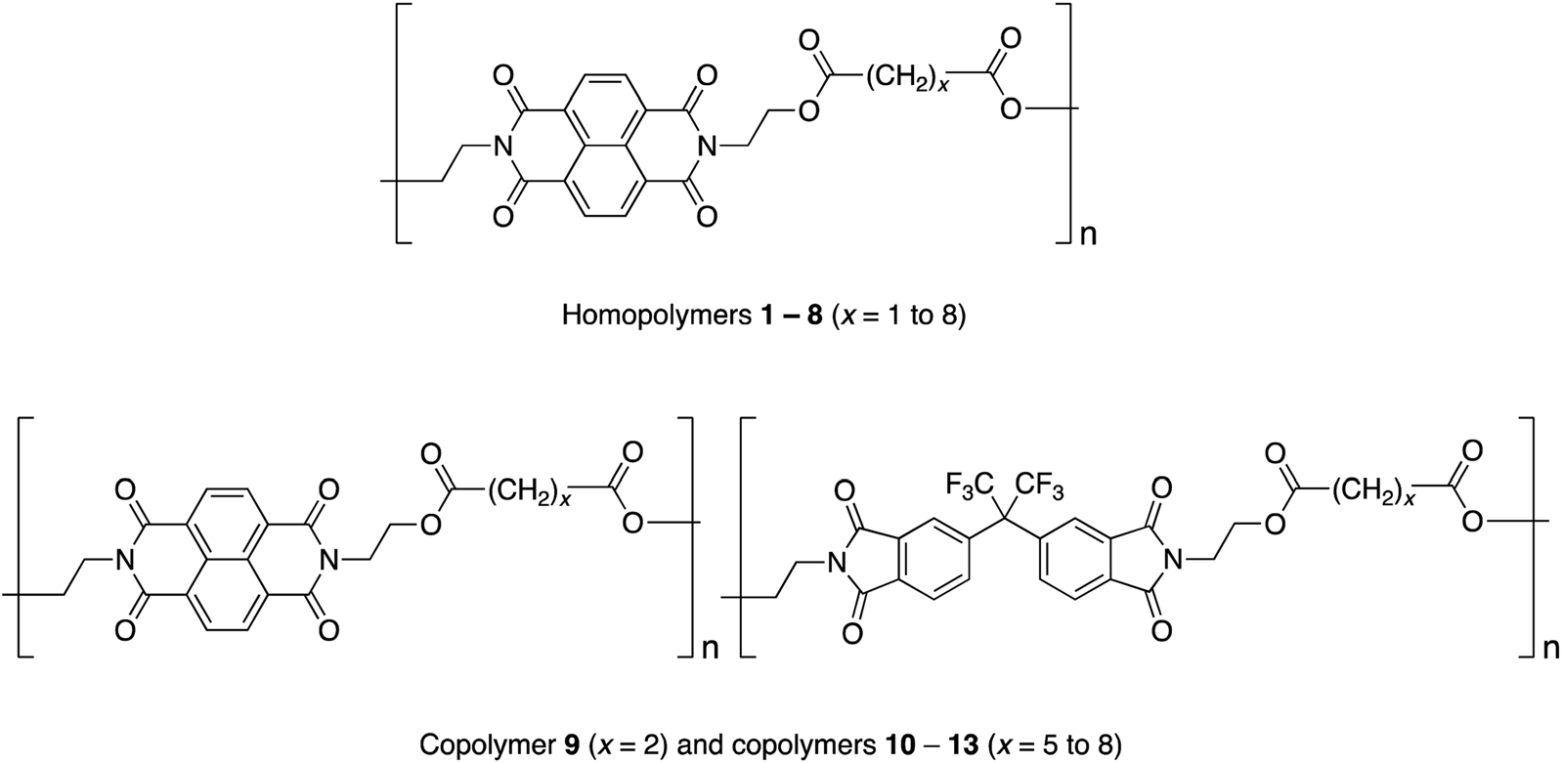
Ester-imide homopolymers and copolymers based on NDI and HFDI.

Studies of homopoly(ester-imide)s containing π-electron-poor NDI residues linked by aliphatic diester units (polymers **1** to **8** in Chart 1) have shown that the strength of supramolecular binding is highly dependent on the length of the diester spacer, with a sharp maximum in binding energy for homopolymer **2**, where *x* = 2.^16^ Computational modelling suggested that the short, “*x* = 2” diester spacer-unit forms a tight chain-fold between two NDI residues that is especially favourable for pyrene binding by intercalation, with close (van der Waals) contact between the complexed aromatic and the two adjacent NDIs.^16^

Experimentally, the pattern of complexation shifts seen for the corresponding 1 : 1 copoly(ester-imide), **9**, (*x* = 2), showed a close similarity to the fractal-based pattern observed previously for the intercalating pyrene complex of a chain-folding NDI–HFDI copoly(ether-imide),^15^ confirming the dual-site binding model (Fig. 1) for this poly(ester-imide).^16^

In the present work we have investigated the complexation of pyrene to NDI/HFDI co-poly(ester-imide)s with longer diester-spacers (Chart 1, copolymers **10** to **13**; *x* = 5 to 8). The long-spacer NDI-based homopolymers **5** to **8** show very much smaller complexation shifts, Δ*δ*, than the tightly chain-folding homopolymer **2**, where *x* = 2.^16^ Nevertheless, in the present work we find that significant upfield complexation shifts of the NDI resonances (up to 0.5 ppm) may still be observed for long-spacer homopolymers and copolymers if sufficiently high concentrations of pyrene (up to 10 equivalents per NDI residue) are used. However, the pyrene complexes of copolymers **10** to **13** (*x* = 5 to 8) show a very different pattern of NDI signals from that observed for the homologous copolymer **9** (*x* = 2), for which intercalative, dual-site binding was established.^16^ The new pattern is consistent with the long-spacer copolymers adopting chain-folds in which single-site binding is preferred but, despite the very different binding mode, analysis of ^1^H NMR data shows that the “last-fraction” type of Cantor set still provides a logical basis for interpreting the observed complexation shifts.

## 2. Experimental section

### 2.1 Materials and instrumentation

Starting materials, monomers, solvents and analytical instrumentation were as described in a previous publication.^16^

### 2.2 Computational methods

Pyrene–NDI complexation energies were obtained using the self-consistent-charge density functional tight-binding (SCC-DFTB) approach, as implemented within the DFTB+ code.^42^ Parameters for all atoms and pairs including elements C, H, N, O were taken from the “mio” parameter set of the Slater–Koster library.^43^ Dispersion corrections based on a Lennard-Jones potential were applied in all simulations.^44^ Simulations of ^1^H NMR spectra were carried out using the “peak table to spectrum” script within Mnova (version 14.1, Mestrelab Research).

### 2.3 Synthesis of copolymer **10** (*x* = 5)


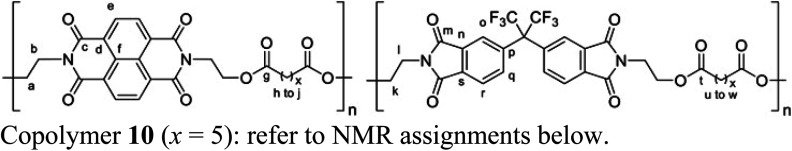
1-Chloronaphthalene (2.5 mL, distilled from calcium hydride), *N*,*N*′-bis(2-hydroxyethyl)-1,4,5,8-naphthalenetetracarboxylic diimide (0.869 g, 2.45 mmol, dried at 100 °C for 24 h), *N*,*N*′-bis(2-hydroxyethyl)hexafluoroisopropylidene-diphthalimide (1.326 g, 2.50 mmol, dried at 100 °C for 24 h) and 1,7-heptanedioyl dichloride (0.999 g, 5.07 mmol) were combined at room temperature and the reaction mixture was then heated at 120 °C for 2 h under a slow nitrogen purge. After cooling to room temperature, the product mixture was dissolved in dichloromethane/hexafluoroisopropanol (4 : 1, v/v, 30 mL) and the solution was added dropwise with stirring into methanol (400 mL). The precipitated copolymer **10** was filtered off, dried at 80 °C for 24 h, purified by three reprecipitations from the above solvent mixture into methanol, and finally filtered off and dried again at 80 °C for 24 h. Yield: 1.63 g, 56%.

Inherent viscosity (*η*_inh_, 25 °C, CHCl_3_/TFE 6 : 1, v/v): 0.61 dL g^−1^. *M*_n_ (from viscosity/GPC calibration: ESI, p. S6†) 22 000. *T*_g_ (DSC): 91 °C. FTIR *ν*_max_ ATR (cm^−1^): 1780 (imide *ν*_C=O_), 1708 (ester *ν*_C=O_), 1389 (imide *ν*_C–N_), 1190 (*vs.*, *ν*_C–F_), 1163 (ester *ν*_C–O–C_). ^1^H NMR (400 MHz, CDCl_3_/TFA 9 : 1, v/v) *δ* 8.75 (s, 4H_e_), 7.93 (d, *J* = 8.0 Hz, 2H_q/r_), 7.84 (s, 2H_o_), 7.77 (d, *J* = 8.1 Hz, 2H_q/r_), 4.58–4.36 (m, 8H_a/b_), 4.37–4.21 (m, 4H_l_), 4.08–3.86 (m, 4H_k_), 2.38–2.11 (m, 8H_h/u_), 1.68–1.42 (m, 8H_i/v_), 1.42–1.11 (m, 6 H_j/w_) ppm. ^13^C NMR (100 MHz, CDCl_3_/TFE 6 : 1, v/v) *δ* 174.65 (C_g/t_), 167.32 (C_m_), 163.14 (C_c_), 139.00 (C_n/s_), 136.01 (C_q/r_), 132.61 (C_p_), 132.25 (C_n/s_), 131.28 (C_e_), 126.78 (C_d/f_), 126.41 (C_d/f_), 124.89 (C_o_), 123.81 (C_q/r_), 61.50 (C_a/k_), 39.51 (C_b_), 37.23 (C_l_), 33.73 (C_h_), 33.66 (C_u_), 28.24 (C_i/v_), 24.09 (C_j/w_) ppm.

Synthetic and characterisation details for the other copolymers reported in this work (**11**, **12** and **13**: *x* = 6, 7 and 8 respectively) are given in the ESI.† The new copolymers were found to be soluble in mixed solvents containing chlorocarbons (CHCl_3_ or CH_2_Cl_2_) and proton-donor solvents such as trifluoroethanol or hexafluoropropan-2-ol, but were insoluble in standard GPC solvents such as THF or DMF. Thus, GPC analyses were not accessible, but a molecular weight calibration based on GPC data (in THF) and viscosity data (in CHCl_3_/trifluoroethanol, 6 : 1 v/v) for the more soluble HFDI-homopoly(ester-imides)^16^ enabled inherent viscosities to be converted to number-average molecular weights (see Section 2.3 above and ESI†).

## 3. Results and discussion

### 3.1 Dual-site or single-site binding?

The NDI-based homopoly(ester-imide)s **1–8** (*x* = 1 to 8) show a marked dependence of pyrene-induced complexation shift on the value of *x*, with the complexation shift showing a sharp maximum at *x* = 2 (Δ*δ* = 0.73 ppm on addition of two equivalents of pyrene per NDI residue). This maximum was shown to result from the presence of a chain-fold geometry between adjacent NDI residues that is particularly favourable for dual-site binding of pyrene.^16^ The very much smaller NDI complexation shifts seen for homopoly(ester-imide)s having more extended spacers (Δ*δ* ≈ 0.2 ppm for *x* = 5 to 8) suggest that, with these polymers, pyrene no longer interacts strongly with the NDI residues *via* intercalative, dual-site binding (Δ*δ* ≈ 0.75 ppm for *x* = 2). In such systems, the lower the pyrene–NDI association constant, *K*_a_, the smaller is the observed NDI complexation shift at a given concentration of pyrene, as the equilibrium position for pyrene-binding is shifted further towards the unbound state. Resonances for monomer residues (HFDI) with no binding affinity for pyrene show zero complexation shifts, even at high concentrations of probe-molecule (see Section 3.5).

A possible alternative to intercalation (1 : 2 binding) is single-site (1 : 1) binding of pyrene to each NDI residue. It is widely recognised – notably in molecular biology – that “multivalent” binding (where a small molecule binds simultaneously to two or more sites on a macromolecule) is a key factor in producing a high association constant.^45^ This would account immediately for the high complexation shifts seen for poly(ester-imide)s **2** and **9** (Chart 1) where 1 : 2 (“divalent”) binding at a tightly chain-folded NDI pair has been established. Conversely the much smaller complexation shifts seen for homologous polymers **5** to **8** and **10** to **13** (Chart 1), with longer spacer-lengths, would be rationalised in terms of a lower association constant for 1 : 1 (“univalent”) binding. Of course there are other factors that can influence the relative magnitudes of association constants, specifically changes in solvation and pre-organisation, but the supramolecular systems under discussion here involve just a single type of small molecule (pyrene) binding to a series of homologous poly(ester-imide)s in a single type of solvent. Solvation and/or pre-organisation contributions to binding should therefore be relatively unchanged between the different systems.

### 3.2 Computational modelling of single-site (univalent) binding

**Fig. 3:**
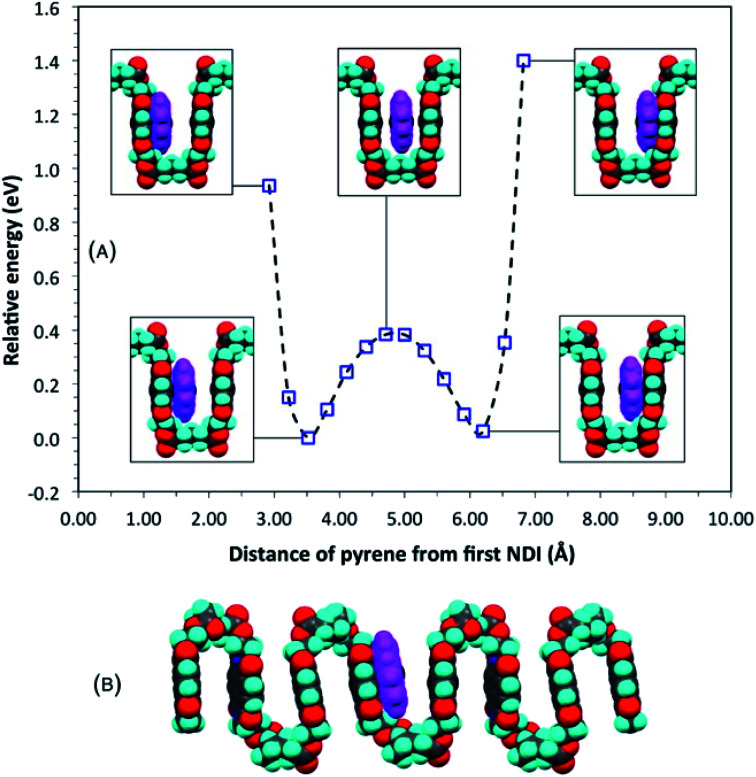
(A) Computed single-point (relative) energies for a model complex of pyrene with homopolymer **5** (*x* = 5) as pyrene is moved across a symmetrical chain fold; (B) energy-minimised model for the same complex. Locating pyrene at the mid-point of the fold, considered for *x* = 5 in an earlier study,^16^ can now be seen to represent only a metastable situation. See ESI† for structure files.

The proposal that single-site binding would be preferred at longer spacer-lengths between NDI residues was tested computationally by constructing an idealised, symmetrical, ester-imide chain-fold with *x* = 5 and inserting a pyrene molecule at one side of the fold, parallel to, but at sub-van der Waals distance (2.92 Å) from, the adjacent NDI residue. To illustrate the potential energy curve as a function of pyrene position, we performed a series of single-point energy calculations, in which the pyrene was moved stepwise across the chain-fold at 0.3 Å steps. The energy of the system fell initially, until the pyrene and NDI residue were just in van der Waals contact, but rose as these components moved apart, and then fell again as the pyrene approached the next NDI along the chain. The double-well potential energy curve (Fig. 3A) shows that single-site binding is indeed strongly favoured. Full minimisation of a seven-residue polymer model with pyrene bound at the central chain-fold confirmed this result, leading to a final structure (Fig. 3B) in which pyrene is located in van der Waals contact with just one NDI residue (centroid–centroid distance = 3.38 Å), and at more than twice this distance (7.24 Å) from the NDI at the other side of the chain-fold.

### 3.3 Numerical analysis of single-site binding

**Fig. 4:**
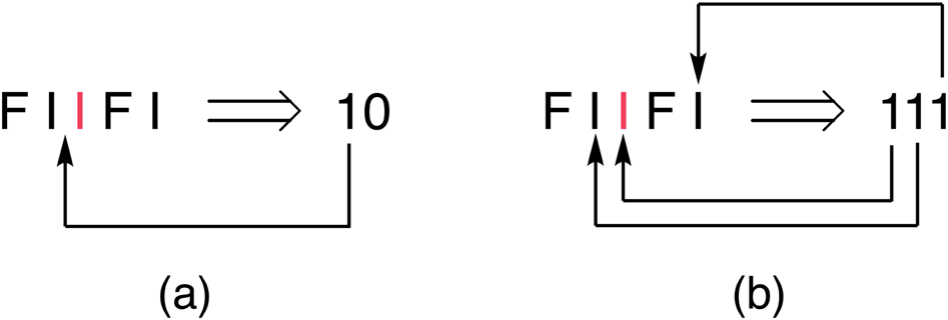
Alternative assignments of shielding codes for the quintet sequence 
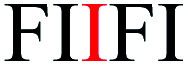
 based on (a) dual-site “intercalative” binding of pyrene at an NDI pair, “II”, or (b) single-site binding at every NDI residue.

The new single-site binding model was then analysed numerically in terms of its predictions for ring-current shielding of the central NDI (“I”) in different sequences within a binary NDI-HFDI (HFDI = “F”) copolymer. This analysis shows that I-centred quintet sequences with single-site binding of pyrene generate three-digit “shielding codes”, rather than the two-digit codes found for dual-site binding. This is because, in single-site binding the central “I” residue is now always an allowed binding site and there are always two further potential binding positions, in each direction, viewed from the centre (Fig. 4b). In the case of dual-site binding, pyrene is only bound when there are two adjacent NDI residues, so that the number and positions of binding sites are more restricted for a sequence of any given length. For example, in the sequence FIIFI, discussed above in a dual-site binding context, there is one central I residue, one adjacent I residue and one next-adjacent I residue, thus generating (for a single-site binding model) the three-digit shielding code 111 (Fig. 4b).

The shielding code is simply a number whose successive digits represent diminishing contributions to the total ring-current shielding experienced by a central, “observed” NDI residue in a comonomer sequence, on complexation by an aromatic molecule. If the “fall-off factor” between digits were 10, the codes would be conventional decimal numbers but in previous work,^15,16^ this factor was found, empirically, to be close to a value of 4, so that the codes can be viewed, at least approximately, as quaternary numbers.

All possible I-centred quintet sequences are enumerated in Table 1, together with their corresponding shielding codes assigned on the basis of single-site binding to “I” residues. Resolution of such sequences in the ^1^H NMR spectrum of a binary I/F copolymer, by complexation of a shielding molecule such as pyrene, should thus afford nine resonances. In the present work, quintets are the longest sequences for which separate ^1^H NMR resonances can be resolved, even at high concentrations of pyrene.

**Table 1:** The sixteen possible “I”-centred quintet sequences in a 1 : 1, random, F/I copolymer. The three-digit “shielding” codes for these sequences are based on single-site binding to I residues. Codes are listed (top to bottom) in order of increasing predicted complexation shift. The degeneracies (*Ω*) are the number of different sequences corresponding to the same code

Code	I-Centred quintet sequences	*Ω*
100	FFIFF	1
101	FFIFI + IFIFF	2
102	IFIFI	1
110	FFIIF + FIIFF	2
111	FFIII + IIIFF + FIIFI + IFIIF	4
112	IFIII + IIIFI	2
120	FIIIF	1
121	FIIII + IIIIF	2
122	IIIII	1

The relative intensities of these resonances correspond to the probability of each sequence occurring in the copolymer, and in a random, 1 : 1 copolymer all sequences of a given length have the same probability. In the present work, integrals measured for a well-resolved group of “triplet level” NDI resonances (copolymer **13**, spectrum S9, ESI page S13†) show relative values of 1.00 : 1.99 : 0.99 and are thus indeed consistent with the intensities predicted for a random copolymer. The relative intensity of a resonance can therefore be predicted directly from the number of different sequences giving rise to the same shielding code, *i.e.* to the degeneracy of that code. Table 1 thus predicts a spectrum, in the NDI region, consisting of nine lines with relative intensities 1 : 2 : 1 : 2 : 4 : 2 : 1 : 2 : 1.

The degeneracy *Ω* of a code can be formally expressed as a function of the code digits *N*_*k*_ (each digit corresponding, in molecular terms, to the occupancy by NDI residues of sites *k* steps away from the centre) as:
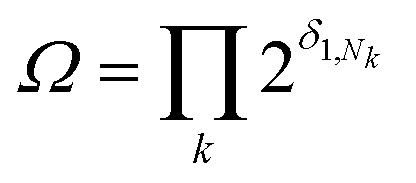
where the symbol 
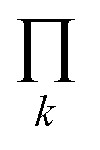
 denotes a product of the argument over values of *k* (the first digit, which is always 1, corresponds to the central I and is not considered in the product), and *δ*_*i,j*_ is the so-called Kronecker delta, defined as:
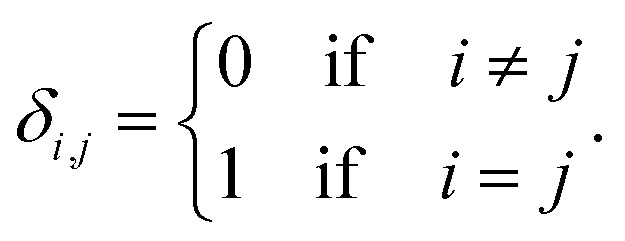


The expression above simply means that the degeneracy doubles for each “1” in the code, because there are two ways of achieving an occupancy of 1, as seen in Table 1. The relative intensities of the peaks are then given by:1
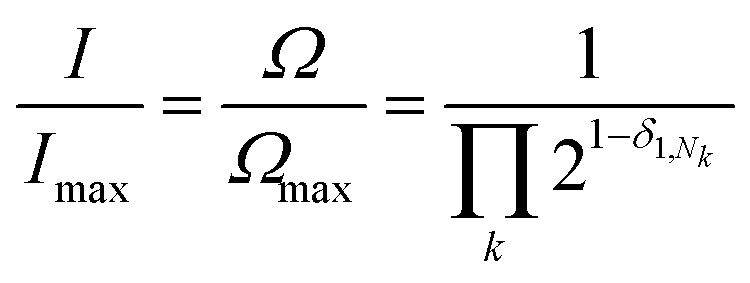


Clearly, the maximum intensity always corresponds to the “central” peak (111 for 3-digit codes – see Table 1) where *N*_*k*_ = 1 for all values of *k*, therefore the degeneracy *Ω* = *Ω*_max_ = 4 and the relative intensity is 1. For all other peaks, the relative intensity is halved for each code-digit different from 1. For example, the peak with code 101 has a relative intensity of 1/2 because it has one digit different from 1, whereas the peak with code 100 has a relative intensity of 1/4 because it has two digits different from 1.

### 3.4 The ^1^H NMR spectrum as a Cantor set

In a high-MW copolyimide, each NDI (“I”) residue may be regarded as being at the centre of a sequence which (in dilute solution) defines its molecular environment. Thus, in NMR terms, we need consider only the resonance arising from the central NDI residue in any sequence: other NDI residues in that sequence are themselves at the centres of other sequences and so are treated separately.

In such systems, complexation of pyrene occurs under fast-exchange conditions on the NMR timescale, so that any atomistic model for the complex can only represent a dynamic, time-averaged structure. Nevertheless, the “chain-folding with intercalation and shielding” model described above (Fig. 1) led directly^15^ to an expression (eqn (2)) that sums the shielding effects, diminishing by a factor *b*, of pyrene binding at successively more distant NDI pairs, up to a maximum value of *k* that is a function of the sequence-length being considered. This summation also takes account of how many pyrene molecules (*N*_*k*_ = 0, 1 or 2) are bound at each type of position in the sequence, viewed from the central NDI residue. For example, the quintet sequence FIIFI has one II-pair adjacent to and including the central I residue, and zero II-pairs at the next-adjacent positions. This sequence thus has only two possible values for *N*_*k*_ (1 and 0) and is assigned the “shielding code” 10. Every possible quintet sequence (see Fig. 6 for experimental reasons why quintet sequences are exemplified here) can similarly be assigned a two-digit code using only the digits 0, 1 and 2, representing input values for *N*_*k*_ in eqn (2). Finally, the sum of shieldings may be scaled by a factor *a* that depends on the molar ratio of pyrene to NDI and on the concentration of NDI residues. The scaling factor reflects an increasing level of ring-current shielding with (i) an increasing overall concentration of the copolymer/pyrene system, where a higher concentration tending to shift the binding equilibrium more towards the bound state, and/or (ii) an increasing molar ratio of pyrene to NDI residues, with a higher ratio leading to a higher proportion of NDI resides being in the bound state. The factor *a* has units of ppm and so enables the otherwise dimensionless total-shielding factor, *T*, to be expressed as a predicted complexation shift for the central, “observed” NDI residue in each sequence.2
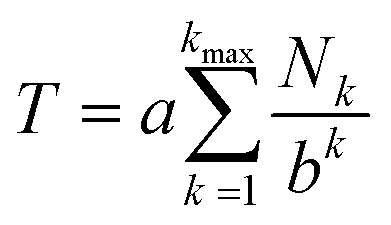


Eqn (2), with *a* = 1, *b* = 4, *N*_*k*_ = 0, 1 or 2, and *k*_max_ = ∞, is the mathematical definition of the fourth-quarter Cantor set.^15^ It should however be noted that, whatever the value of *b* (>3, integral or non-integral), this type of equation represents a fractal set obtained by summing an exponential-decay series. Although *a* does change between spectra, due to the change in pyrene concentration, it is a constant for each individual spectrum, and each spectrum can therefore be predicted from eqn (2). Since fractals are scale-invariant, the introduction of the factor *a* does not affect the fractal nature of the system. The binding model shown in Fig. 1 thus predicts a fractal distribution of ring-current shieldings for all possible sequences in a binary copolyimide.

**Fig. 5:**
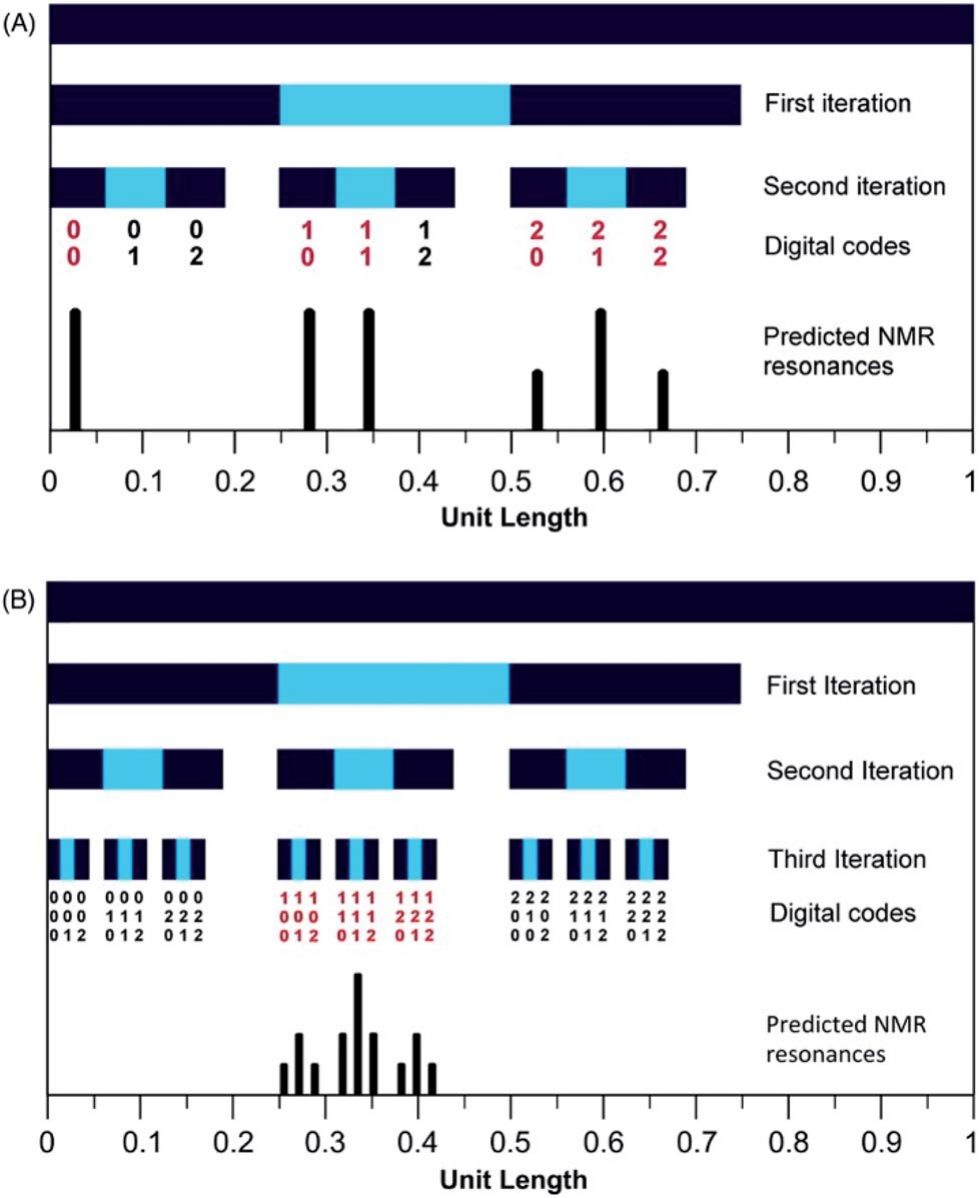
Graphical construction of the fourth-quarter Cantor set. This procedure involves dividing a line of unit length into four equal parts, discarding the fourth quarter, and repeating these operations on the remaining three segments, through an infinity of iterations. The construction is equivalent to eqn (2) when *a* = 1, *b* = 4, *N*_*k*_ = 0, 1 or 2, and *k*_max_ = ∞. In construction (A) the dual-site binding model results in prediction of a widely-spaced six-line spectrum (see ref. 15 for experimental confirmation of this pattern) whereas in construction (B) the single-site model predicts a narrowly-spaced nine-line spectrum. In both cases the possible quintet sequences generate only a sub-set (codes shown in red) of the complete Cantor set, but these subsets differ markedly as a result of the change in binding model. *Note*: in this figure, shielding codes are read vertically downwards.

In the present system, relative complexation shifts of the predicted resonances could be calculated using the fourth-quarter Cantor set (eqn (2) and Fig. 2). However, eqn (2) emerged specifically from a tightly chain-folded, dual-site binding model,^15^ and it was by no means certain that it would remain valid for a single-site binding system. Specifically, there was no obvious reason why the factor *b*, representing the fall-off factor of ring-current shielding with the numerical position *k* (adjacent = 1, next-adjacent = 2, next–next adjacent = 3 *etc.*) of a pyrene binding site relative to the observed NDI residue, should remain close to a value of four when the positions of binding sites further out along the chain from the central NDI residue (previously defined as two-NDI intercalation sites) are now defined just as single NDI residues. However, to allow direct comparison of the predictions from dual-site and single-site binding models, eqn (2) (with *a* = 1 and *b* = 4) was applied to both models, with the results for quintet sequences shown in Fig. 5. Here the graphical construction of the fourth quarter Cantor set^15,16^ allows a prediction of the NDI resonance-patterns for both binding models.

Using an integral exponential fall-off factor (*b* = 4) enables immediate visualisation of the type of fractal involved (a last-fraction Cantor set) *via* the graphical construction shown in Fig. 5, but there is no theoretical requirement for the fall-off factor *b* to be exactly 4. We have shown previously^15^ that *b* can take any value, including non-integral values, greater than 3 without affecting the fractal character of eqn (2) (the corresponding fractal dimension is *D* = ln(3)/ln(*b*): see Section 9 of the ESI† in ref. 15). However, the present analysis (see below) does suggest that the experimental value of *b* for copolymer **10** is (i) a constant, independent of pyrene concentration, and (ii) close to 4.

In Fig. 5, construction (A), after two iterations, generates all the possible two-digit shielding codes for I/F quintet sequences on the basis of dual-site (pairwise) binding of pyrene, and gives a predicted pattern for the corresponding ^1^H NMR resonances of NDI protons.^16^ Three potential shielding codes [01, 02 and 12, shown in black in construction (A)] are not generated by any I-centred quintet sequence on the basis of dual-site binding, and so no corresponding resonances are predicted for those codes. Construction (B) of Fig. 5 generates, after three iterations, all possible three-digit codes for I/F quintets based on the single-site binding model. Here, only codes beginning with the digit 1 emerge from single-site binding to I-centred quintet sequences (Table 1), as the central I residue is now always a binding site for pyrene. Unused codes are again shown in black.

**Fig. 6:**
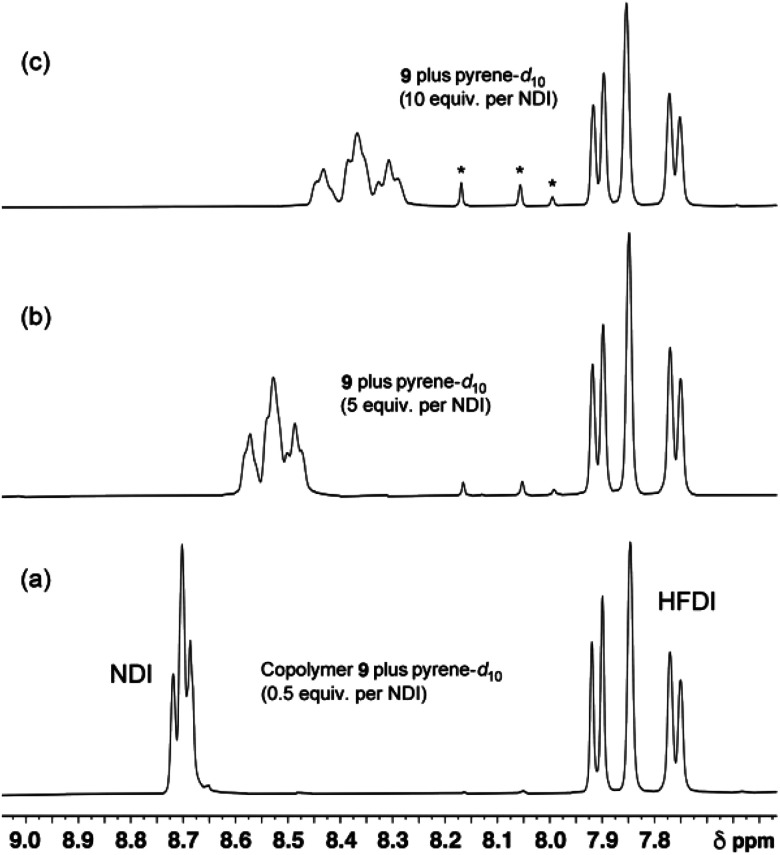
^1^H NMR spectra of copolymer **10** (4 mM in NDI residues in CDCl_3_/trifluoroethanol, 6 : 1 v/v) in the presence of increasing levels of pyrene-*d*_10_. At low levels of pyrene (0 to 3 equivalents per NDI) only three NDI resonances can be identified. These are assigned to the I-centred triplets FIF, FII/IIF and III which, at these low pyrene concentrations, are the only resolvable I-centred sequences. At higher pyrene concentrations however, nine resonances are resolved, and these are assigned to the nine groups of quintet sequences shown in Table 1. Starred resonances represent residual pyrene protons in the (99.8%) deuterated pyrene. Further details of this titration, together with analogous titration data for copolymers **11**, **12** and **13**, are given in the ESI.†

Although true mathematical fractals are valid across all length scales, in the physical world objects showing fractal character invariably display self-similarity over only a small number of different length scales. Indeed, it is generally considered that real objects are “described as fractal if they contain parts that, at two or more smaller scales, appear in some way similar to the whole”.^34^ The experimental data (Section 3.5) do indeed provide evidence for self-similarity over only a small number of length scales, corresponding to three iterations of the mathematical construction (Fig. 5B and 6): copolymer systems showing more highly resolved NMR peak-separations are clearly needed to test the present fractal model further.

### 3.5 Experimental evidence for single-site binding

The patterns of NDI resonances predicted in Fig. 5 are very different for the two different binding modes, even though exactly the same set of sixteen I-centred quintet sequences (Table 1) are involved. A ^1^H NMR titration of copolymer **10** against pyrene-*d*_10_ (0.5 to 10 equivalents per NDI residue) is shown in Fig. 6. The predictions of Table 1 and Fig. 5B are clearly vindicated, in that the final spectrum (Fig. 6c) shows nine NDI resonances whose relative intensities agree, at least approximately, with the values (1 : 2 : 1 : 2 : 4 : 2 : 1 : 2 : 1) predicted from single-site binding to a random 1 : 1 copolymer.

**Fig. 7:**
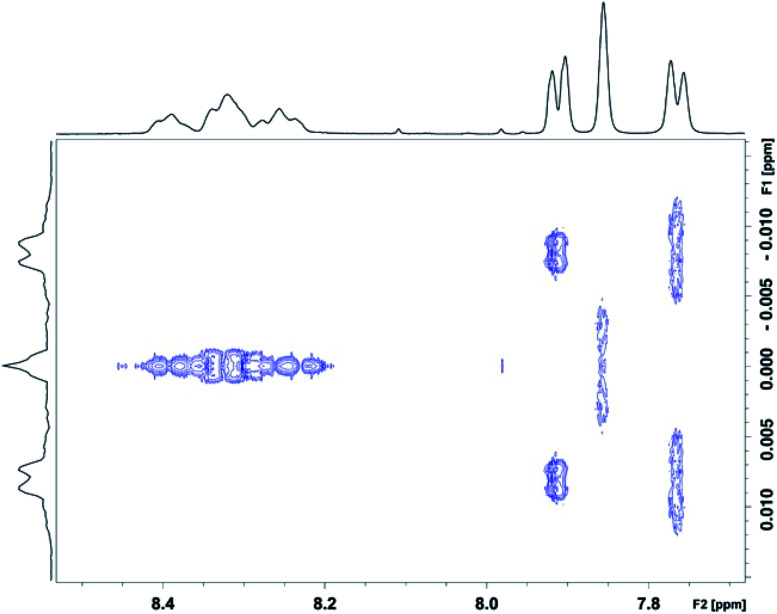
JRES spectrum showing homonuclear (^1^H–^1^H) couplings (vertical axis) in the diimide region for copolymer **10** in the presence of 8 mole equivalents of pyrene-*d*_10_ per NDI residue. The HFDI resonances are found between 7.7 and 8.0 ppm, and the (sequence-dependent) NDI resonances between 8.2 and 8.5 ppm. The 2D-JRES spectrum was run in (CDCl_3_/trifluoroethanol, 6 : 1 v/v) at a copolymer concentration of 4 mM in NDI residues.

In order to check that the nine ^1^H NMR lines observed at high pyrene : NDI ratios (Fig. 6c) are indeed single NDI resonances resulting from resolution of the nine sequence-groups identified by shielding code in Table 1, and do not result from spin–spin coupling (which could potentially arise for *ortho*-related protons an NDI residue at the centre of an unsymmetrical sequence), we next carried out a 2D-JRES analysis of the diimide region of the ^1^H NMR spectrum (Fig. 7). This type of spectrum plots *J* values against associated δ values and enables the magnitudes of all *J*–*J* couplings in the spectrum to be determined.

The JRES result is quite clear: *ortho*, *meta* and *para* couplings for the HFDI resonances are obviously identifiable, having *J*-values (at 400 MHz) of 0.0170, 0.0040 and 0.0015 ppm respectively, and the maximum coupling in the NDI region of the JRES spectrum is *ca.* 0.0010 ppm. This latter *J*-value is negligible when compared to the separation of the various NDI resonances in Fig. 6c, for which the minimum value is *ca.* 0.02 ppm (a 20-fold difference): spin–spin coupling thus plays no part in generating the NDI resonance-pattern.

Interestingly, as shown in Fig. 6, the ^1^H resonances associated with non-binding HFDI residues are essentially unaffected by the presence of pyrene, even at the highest concentration. From the point of view of a “static” physical model it might be expected that neighbour-bound pyrenes would exert similar degrees of ring-current shielding on both NDI and HFDI residues. The fact that they do not indicates that the dynamics of the system (fast exchange on the NMR timescale between different copolymer chain-conformations; fast exchange between bound and unbound pyrene molecules; and the possibility of rapid diffusion of pyrene between binding sites) must be taken into account if a successful atomistic model is to be developed. This would go well beyond the scope of the present work, but a possible approach is suggested by a recent report that, in certain DNA molecules, the binding affinity of a “central” recognition site for a specific protein is strongly amplified by DNA regions flanking the recognition site. These regions contain long tracts of degenerate recognition-sites which appear to function as “antennae” that attract molecules of the protein to the central binding site through exchange among neighbouring binding sites.^46,47^

We next set out to evaluate the “fall-off factor”, *b*, for shielding under single-site binding conditions experimentally, rather than empirically. Eqn (2) was therefore generalised and expanded, taking the shielding by pyrene bound directly to the central NDI (*T*_0_) out of the summation, as this shielding is always present whatever the sequence under consideration (eqn (3)). This operation also has the effect of transforming the summation term (for *a* = 1 and *k* = 1 to ∞: *i.e.* when the I-centred copolymer sequence is infinitely long) from a subset of the fourth-quarter Cantor set (Fig. 5B) into the complete set. Even though spectroscopic resolution in the present system limits consideration to only quintet sequences, for which the resulting equation sums only over the range *k* = 1 to 2, the rapid fall-off in shielding as pyrene molecules are bound further out from the “observed” NDI residue leads to rapid convergence of the predicted resonance-pattern at physically-meaningful linewidths.3
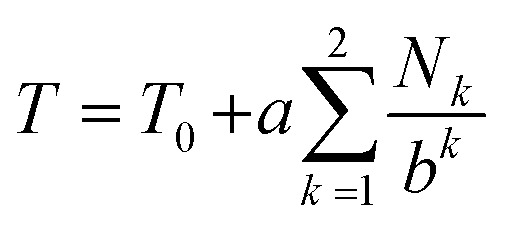


**Fig. 8:**
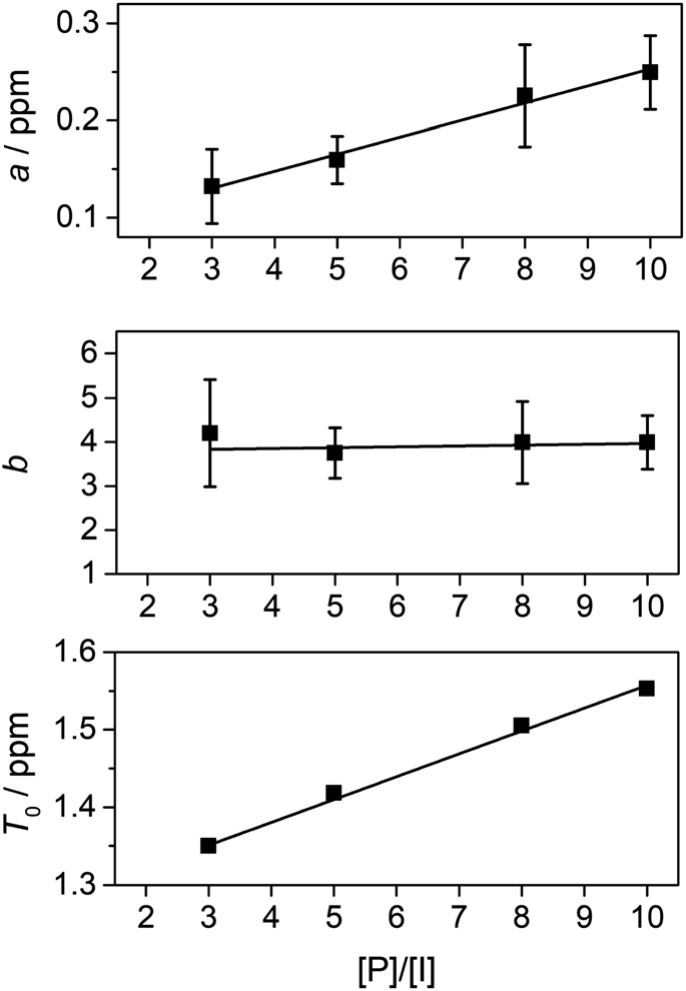
Variation of parameters *a*, *b* and *T*_0_ with the molar ratio of pyrene-*d*_10_ to NDI residues in copolymer **10**. The value of *b* is essentially constant. The equations of fit for *a* and *T*_0_ are: *a* = 0.0175([*P*]/[*I*]) + 0.0776, and *T*_0_ = 0.029([*P*]/[*I*]) + 1.268. Error bars in the *T*_0_ plot are not visible because they are smaller than the symbol size.

We were then able to determine experimental values for the parameters *a*, *b* and *T*_0_ by fitting complexation shifts from the ^1^H NMR titration of copolymer **10** (*x* = 5) to eqn (3) (see ESI† for full details of the fitting procedure). This analysis showed that *a* and *T*_0_ both vary in an approximately linear way with the concentration of pyrene-*d*_10_, but that *b* is independent of pyrene concentration and remains constant, within experimental error, at a value close to 4 (Fig. 8). This result confirms that the observed pattern of NDI resonances seen in Fig. 5c does indeed reflect the distribution of ring-current shieldings predicted from the fourth-quarter Cantor set. However, it is important to note that currently we can only verify the exponential fall-off with that value of *b* over the first two steps of the model; contributions at longer distances cannot be resolved directly. We therefore cannot rule out that the fall-off factor *b* might change at higher *k*, which would require a more complicated model to describe the spectrum. Our fractal model (implying a constant *b* over all steps) is thus the simplest model that can be used to explain the current data.

### 3.6 ^1^H NMR titrations of pyrene-*d*_10_ with copolymers **10**, **11** and **12**

**Fig. 9:**
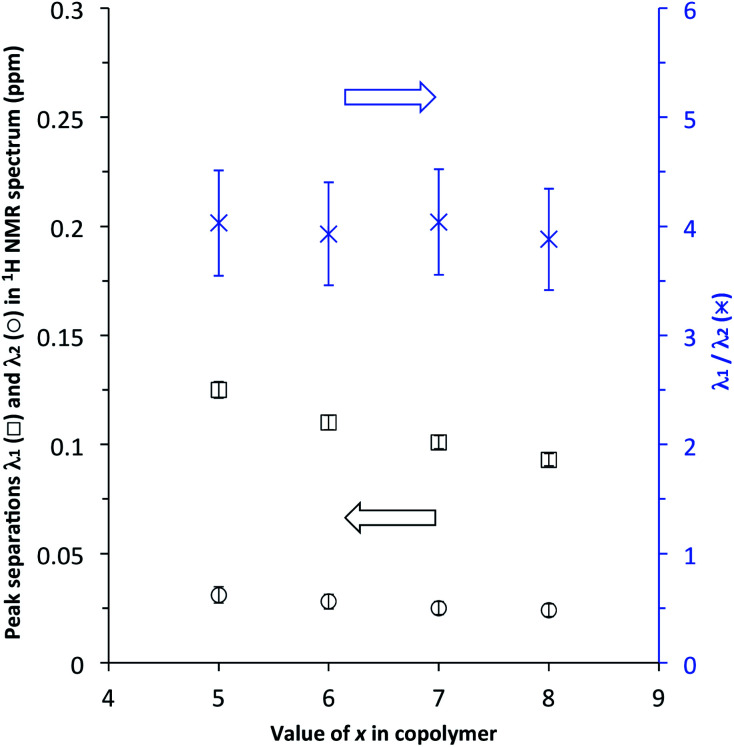
Fall-off in NDI peak separations *λ*_1_ and *λ*_2_ (see ESI†) in the ^1^H NMR spectra of copolymers **10**, **11**, **12** and **13** (*x* = 5, 6, 7 and 8 respectively) at [*P*]/[*I*] = 10. Note that, although *λ*_1_ and *λ*_2_ both diminish with increasing spacer-length *x*, the ratio *λ*_1_/*λ*_2_ is (within error) essentially constant.

The pattern of NDI resonances observed for copolymer **10** (*x* = 5) with increasing pyrene concentration (Fig. 6) was also found for the longer-spacer copolymers synthesised in this work (copolymers **11**, **12** and **13**, where *x* = 6, 7 and 8 respectively). Full titration data for these copolymers are given in the ESI.† The predicted pattern of intensities (1 : 2 : 1 : 2 : 4 : 2 : 1 : 2 : 1) at [*P*]/[*I*] = 10 is again evident, but measurements of the line-spacings *λ*_1_ and *λ*_2_ (see ESI†) show that there is a small but consistent fall-off in these as the spacer-length increases (Fig. 9). This is consistent with pyrene molecules being bound progressively further out from the central “observed” NDI residue in any particular sequence, with a consequent reduction in the long-range ring current shielding that, in NMR terms, differentiates one sequence from another. It may be noted, however, that the shielding fall-off factor *b* (eqn (3)), measured as the ratio of *λ*_1_ to *λ*_2_, remains close to a value of 4, as also found for other, related copolymer systems.^15,16^ Note however that *b* could, in principle, take any value ≥3 (including non-integer values) without affecting the fractal character of eqn (3).^15^

### 3.7 Simulation of the ^1^H NMR titration between pyrene-*d*_10_ and copolymer **9**

**Fig. 10:**
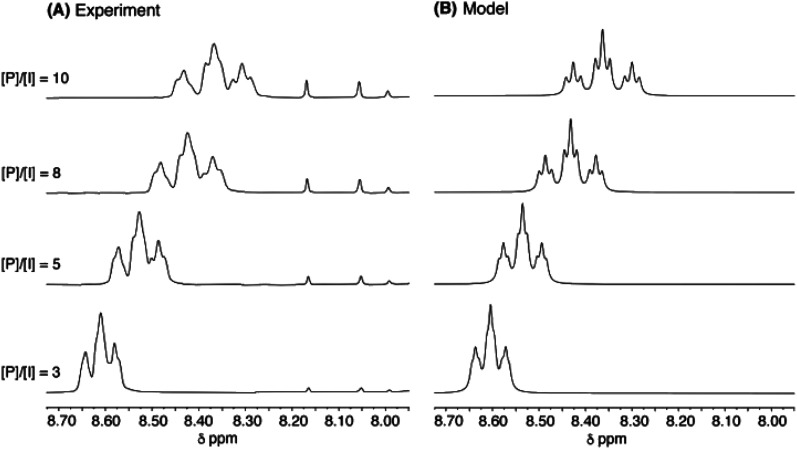
(A): Experimental data for titration of copolymer **10** against pyrene-*d*_10_ in CDCl_3_/trifluoroethanol (6 : 1, v/v). [*P*]/[*I*] = molar ratio of pyrene to NDI residues. Signals between 7.90 and 8.20 ppm correspond to residual protons in pyrene-*d*_10_. (B): simulated data from eqn (1) and (3), based on quintet sequences and using parameters *a*, *b* (= 4) and *T*_0_ as derived above, at a constant linewidth of 4 Hz.

As a final test of the validity of eqn (1) (for relative intensities) and eqn (3) (for complexation shifts) across a range of pyrene concentration, the titration of copolymer **10** with pyrene was simulated for quintet sequences, at four different [*P*]/[*I*] ratios, using the parameters *a*, *b* and *T*_0_ derived as shown in Fig. 8. The resulting simulation (4 Hz linewidth) is shown in Fig. 10B, where it is compared with the corresponding experimental data. The comparison is very close, although the simulation shows somewhat better signal-resolution at high [*P*]/[*I*] ratios.

**Fig. 11:**
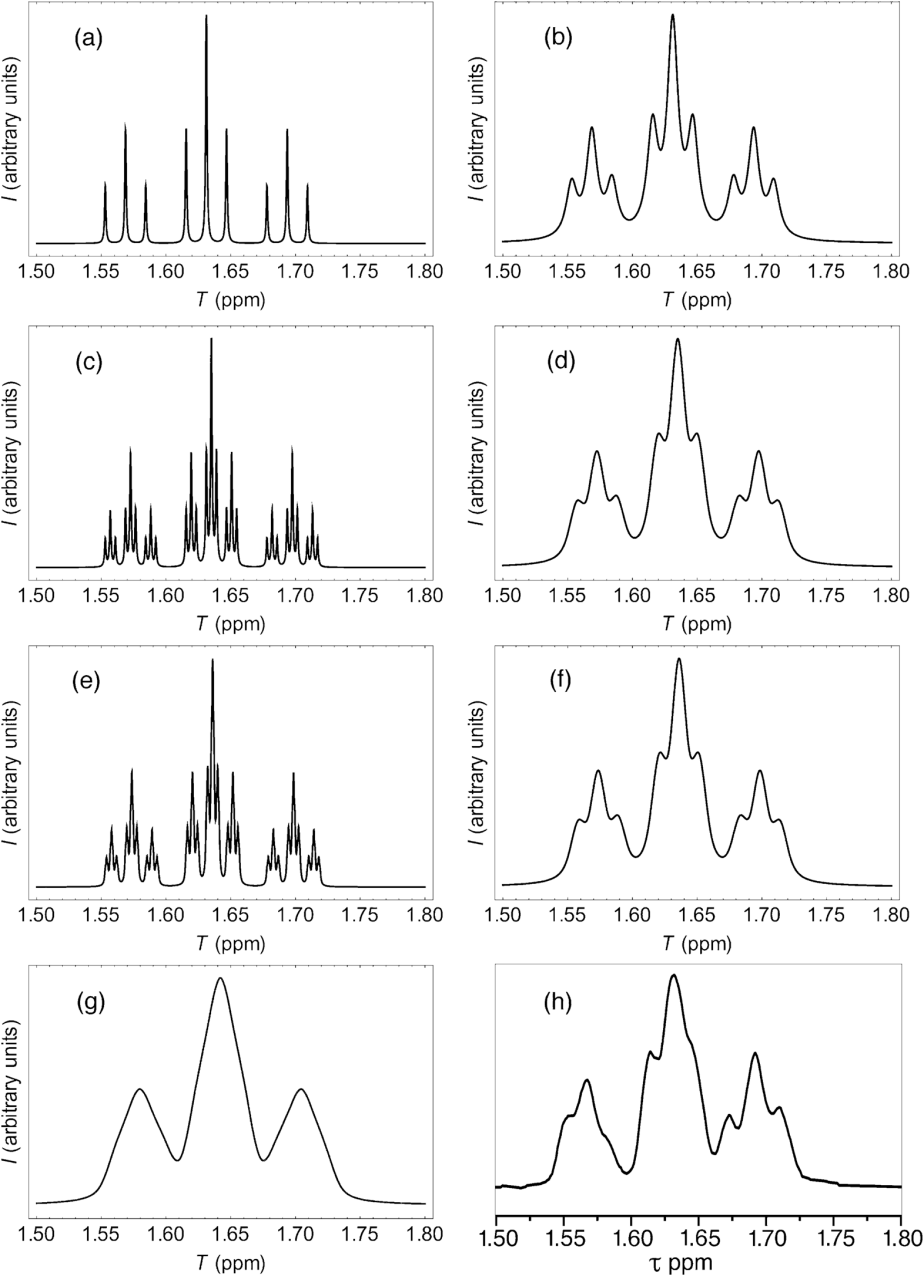
(a) Predicted NDI resonance pattern (0.5 Hz linewidth) for the ^1^H NMR spectrum of copolymer **10** at 10 mol equiv. of pyrene, using the exponential-decay model (eqn (3)) for quintet sequences (*k*_max_ = 2, nine resonances); (b) As (a) but simulated at 4 Hz linewidth; (c) As (a) but for septet sequences (*k*_max_ = 3, twenty-seven resonances); (d) As (c) but simulated at 4 Hz linewidth; (e) As (a) but for nonet sequences (*k*_max_ = 4, eighty-one resonances); (f) As (e) but simulated at 4 Hz linewidth; (g) As (f) but simulated from a quadratic-decay model; (h) experimental spectrum.

More rigorous simulations using longer sequence-lengths (septets and nonets) reproduced the experimental NMR signals rather better than the quintet simulation, as a result of the emergence of additional (fractal) fine structure in these further iterations of the fourth-quarter Cantor set. The effect is illustrated in Fig. 11 for [*P*]/[*I*] = 10, where the “true” structures of the simulated spectra [(a), (c) and (e)] are evident at a linewidth of 0.5 Hz (0.00125 ppm at 400 MHz), and the “observed” patterns [(b), (d) and (f)] are generated using a more physically-realistic linewidth of 4 Hz. Note that the “observed” pattern essentially converges beyond the septet level, because eqn (3) produces a rapid, exponential decay of calculated ring-current shielding with the distance of the binding site from the central NDI residue.

It could be argued, *a priori*, that a quadratic decay of ring-current shielding with distance (analogous to the quadratic decay of a simple magnetic field) might be expected. Indeed, if we consider just a quintet sequence, the distance of the “observed” NDI residue from the second-adjacent NDI is twice the distance from the first-adjacent NDI, which on a quadratic-decay model would give a fall-off in shielding by a factor of 2^2^ = 4, exactly as in the exponential (fractal) model. However, including longer sequence-lengths results in a much slower decay (×1/4, ×1/9, ×1/25 *etc.*) than in the exponential model, where the corresponding values are (×1/4, ×1/16, ×1/64 *etc.*). As a consequence, the higher-order resonances predicted by the quadratic model tend increasingly to “fill the gaps” between the lower-order signals, resulting in complete loss of the fine structure that is observed experimentally. This is illustrated in Fig. 11, where the “nonet” spectrum predicted from the exponential-decay model [11(f)] is compared to that from a quadratic model [11(g)], and both are compared to the experimental spectrum [11(h)]. Full details of these simulations are given in the ESI.†

It also proved possible to test the quadratic model against the exponential model by reference to the “dual-site binding” data reported in ref. 15. Here the experimental spectrum is sufficiently well-resolved to show the effects of including septet, rather than just quintet sequences in the analysis. As shown in the ESI (Fig. S13†), comparisons of the predictions from both models with the experimental data strongly favour the original, exponential model.

## 4. Conclusions

As the length of the diester spacer-unit between NDI residues in poly(ester-imide)s increases, the mode of supramolecular binding between pyrene and NDI changes from “dual-site”, *i.e.* intercalation between two adjacent diimide residues linked by a sharp chain-fold, to “single-site” where each pyrene binds to just one NDI unit, with the polymer chain folding much more loosely. Nevertheless, the ^1^H NMR spectrum of the new 1 : 1 bound copolymer system retains the fractal character observed previously in the 1 : 2 binding system, although showing a different resonance pattern. From this, and results reported in an earlier paper,^16^ we show that a simple mathematical model, based on fractal geometry, describes well both scenarios. Computational modelling indicates that single-site binding is strongly preferred for at least one specific “long” spacer, and ^1^H NMR titrations of NDI/HFDI copolymers against pyrene show a pattern of NDI resonances emerging at high pyrene concentrations that shows distinct fractal character in terms of chemical shift. A detailed analysis of the titration data shows that the underlying mathematical fractal is a last-fraction Cantor set, and that the relative intensities of the observed resonances correspond to the number of different quintet sequences contributing to each resonance. Although the specific last-fraction involved (approximately one quarter) has been identified experimentally only from a resolution-limited range of NMR data, it has been shown by simulation that the observed NDI resonance-pattern is fully consistent with a mathematical model involving exponential decay of ring-current shielding by pyrene binding at neighbouring NDI binding sites. There are clear challenges for future research in developing a fully atomistic model that can account for this result, and in identifying novel copolymer/probe-molecule systems that afford more highly resolved ^1^H NMR spectra.

## Conflicts of interest

There are no conflicts of interest to declare.

## Supplementary Material

SC-011-D0SC03730C-s001

SC-011-D0SC03730C-s002

SC-011-D0SC03730C-s003

SC-011-D0SC03730C-s004

SC-011-D0SC03730C-s005

SC-011-D0SC03730C-s006

SC-011-D0SC03730C-s007
