# Supplementary material for: Single-site binding of pyrene to poly(ester-imide)s incorporating long spacer-units: prediction of NMR resonance-patterns from a fractal model
Source: Chem Sci. 2020 Oct 9;11(44):12165–77. doi: 10.1039/d0sc03730c (PMC8162740; doi:10.1039/d0sc03730c)
Supplement: SC-011-D0SC03730C-s007 [file SC-011-D0SC03730C-s007.pdf]

Electronic Supplementary Material (ESI) for Chemical Science.
This journal is © The Royal Society of Chemistry 2020

Single-site binding of pyrene to poly(ester-imide)s incorporating long spacer-units: Prediction of NMR resonance-patterns from a fractal model

Marcus Knappert,[a] Tianqi Jin,[a] Scott D. Midgley,[a] Guanglu Wu,[b] Oren A. Scherman,[b] Ricardo Grau-Crespo[a] and Howard M. Colquhoun[a*]

[a] *Department of Chemistry, University of Reading, Whiteknights, Reading, RG6 6AD, UK*

[b] *Melville Laboratory for Polymer Synthesis, Department of Chemistry, University of Cambridge, Lensfield Road, Cambridge CB2 1EW, UK*

ELECTRONIC SUPPORTING INFORMATION

Information	Page
Materials and instrumentation	S3
Computational methods	S3
Synthesis and characterisation of NDI-HFDI 1:1 copoly(ester imide)s	S4
**Figure S1**. Calibration plot: Inherent viscosity vs M_n (GPC)	S7
**Figure S2**. ^{1}H and ^{13}C NMR spectra of copolymer **10**	S8
**Figure S3**. ^{1}H and ^{13}C NMR spectra of copolymer **11**	S9
**Figure S4**. ^{1}H and ^{13}C NMR spectra of copolymer **12**	S10
**Figure S5**. ^{1}H and ^{13}C NMR spectrum of copolymer **13**	S11
Protocol for ^{1}H NMR titration at constant concentration of NDI	S12
**Figure S6**. ^{1}H NMR titration of copolymer **10** against pyrene-d_{10}	S12
**Figure S7**. ^{1}H NMR titration of copolymer **11** against pyrene-d_{10}	S13
**Figure S8**. ^{1}H NMR titration of copolymer **12** against pyrene-d_{10}	S13
**Figure S9**. ^{1}H NMR titration of copolymer **13** against pyrene-d_{10}	S14
Equation-fitting analysis	S15
**Figure S10**. Variation of λ_1 and λ_2 with the molar ratio of pyrene to NDI	S16
An alternative, quadratic decay model: why it is not satisfactory	S17

* Email: h.m.colquhoun@rdg.ac.uk

Figure S11. Simulated ^{1}H NMR spectra (0.5 Hz linewidth) based on the alternatives of exponential or quadratic decay S18

Figure S12. Simulated ^{1}H NMR spectra (4 Hz linewidth) based on exponential or quadratic decay, and comparison with the experimental data. S19

Figure S13. Comparison of predictions from the exponential and quadratic models with experimental ^{1}H NMR data published in an earlier paper.[‡] S20

Methodology and calculation tables for ^{1}H NMR simulations S21

Electronic data files

Atomic coordinates for polymer/pyrene complexes (models shown in Figure 2) as electronic files in .mol2 format.

‡ Elements of fractal geometry in the ^{1}H NMR spectrum of a copolymer intercalation-complex. J.S. Shaw, R. Vaiyapuri, M.P. Parker, C.A. Murray, K.J.C. Lim, C. Pan, M. Knappert, C.J. Cardin, B.W. Greenland, R. Grau-Crespo and H.M. Colquhoun. *Chem. Sci*., **2018**, *9*, 4052-4061.

Materials and Instrumentation

Starting materials, solvents, diacyl chlorides $ClCO(CH_2)_xCOCl$ (x = 5 to 8) were purchased from Sigma-Aldrich, ThermoFisher or Fluorochem and were used as received. The diimide-based diols *N,N′*-bis(2-hydroxyethyl)hexafluoro-isopropylidene-diphthalimide (**1**) *N,N′*-bis(2-hydroxyethyl)-pyromellitimide (**2**) and *N,N′*-bis(2-hydroxyethyl) naphthalene-1,4,5,8-tetracarboxylic diimide (**3**) were synthesised as described in a previous publication.[S1] Proton and ^{13}C NMR spectra were recorded on a Bruker Nanobay 400 spectrometer (400 MHz for ^{1}H and 100 MHz for ^{13}C NMR) and on a Bruker 400 AVANCE III Smart Probe spectrometer at 298 K. Proton chemical shifts (δ) are reported in ppm relative to tetramethylsilane (TMS, δ = 0.00 ppm), referred to residual ^{1}H solvent peaks, and ^{13}C NMR chemical shifts are similarly reported relative to TMS. Fourier transform infrared (FTIR) spectra were recorded on a Perkin Elmer 100 Spectrum FT-IR using a diamond ATR sampling accessory. Phase transitions were identified by DSC under nitrogen, using a TA Instruments DSC 2920 instrument, at a scan rate of 10 °C min^{-1}. Inherent viscosities (η_{inh}) were measured at 25 °C with 0.1% w/v solutions of polymer in chloroform/trifluoroethanol (6:1, v/v) using a Schott Instruments CT 52 auto-viscometer with glass capillary No. 53103.

Computational methods

Pyrene binding (intercalation) energies were obtained using the self-consistent-charge density functional tight-binding (SCC-DFTB) approach, as implemented within the DFTB+ code.[S2] Parameters for all atoms and pairs including elements C, H, N, O were taken from the “mio” parameter set of the Slater-Koster library.[S3] Dispersion corrections based on a Lennard-Jones potential were applied in all atomistic simulations.[S4] Simulations of ^{1}H NMR spectra were carried out using the "peak table to spectrum" script within *Mnova* (version 14.1, Mestrelab Research, Santiago de Compostela).

Synthesis and characterisation of NDI-HFDI 1:1 copoly(ester imide)s

Copolymer 11 (*x* = 6):

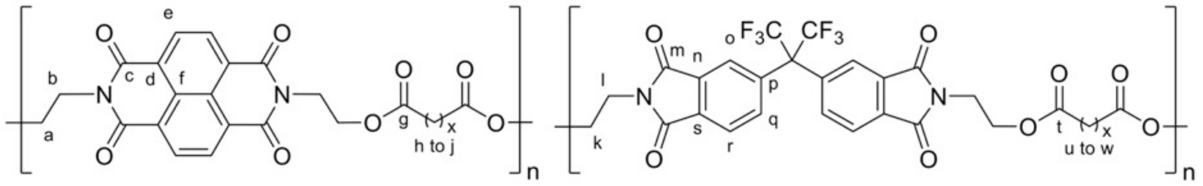

N,N'-Bis-(2-hydroxyethyl)-1,4,5,8-naphthalenetetracarboxylic diimide (0.6059 g, 1.71 mmol), *N,N'*-bis(2-hydroxyethyl)-hexafluoroisopropylidene-diphthalic diimide (0.9224 g, 1.73 mmol), and octanedioyl dichloride (0.7309 g, 3.46 mmol) were dissolved in anhydrous 1-chloronaphthalene (2.0 mL) and the mixture was heated with stirring under a slow nitrogen purge to 130 °C, resulting in evolution of HCl which continued for 15 min. After a total of 45 min. at 130 °C, the reaction was cooled to room remperature and the product mixture was taken up in a mixture of dichloromethane and trifluoroethanol (4:1, v/v; 30 mL). The solution was added dropwise with stirring to methanol (400 mL) and the precipitated copolymer was filtered off, washed with methanol, dried under vacuum at 80 °C and finally purified by two further reprecipitations from the same solvent mixture. Yield 1.85 g (92%).

Inherent viscosity (η_{inh}, 25 °C, $CHCl_3$/TFE 6:1, v:v): 1.26 dL g^{-1}. M_n (from viscosity/GPC calibration; p. S6) 46,000. T_g (DSC): 79 °C. FTIR ν_{max} ATR (cm^{-1}): 1780 (imide νC=O), 1704 (ester νC=O), 1387 (imide νC-N), 1189 (vs, νC–F), 1164 (ester νC-O-C). ^{1}H NMR (400 MHz, $CDCl_3$/TFA 9:1 v:v) δ 8.84 (s, 4H_e), 7.99 (d, *J* = 7.8 Hz, 2$H_{q/r}$), 7.94–7.79 (m, 4$H_{q/r/o}$), 4.74–4.47 (m, 8$H_{a/b}$), 4.47–4.29 (m, 4H_l), 4.16–3.91 (m, 4H_k), 2.46–2.23 (m, 8$H_{h/u}$), 1.63–1.41 (m, 8$H_{i/v}$), 1.41–1.13 (m, 8$H_{j/w}$). ^{13}C NMR (100 MHz, $CDCl_3$/TFE 6:1, v:v) δ 174.81 ($C_{g/t}$), 174.65 ($C_{g/t}$), 167.25 (C_m), 163.13(C_c) , 139.00 ($C_{n/s}$), 135.98($C_{q/r}$), 132.61(C_p) , 132.25 ($C_{n/s}$), 131.25 (C_e), 126.78 ($C_{d/f}$), 126.41($C_{d/f}$), 124.87 (C_o), 123.80 ($C_{q/r}$), 61.45 ($C_{a/k}$), 39.52 (C_b), 37.24 (C_l), 33.90 (C_h), 33.82 (C_u), 28.49 ($C_{i/v}$), 24.28 ($C_{j/w}$) ppm.

Copolymer 12 (*x* = 7):

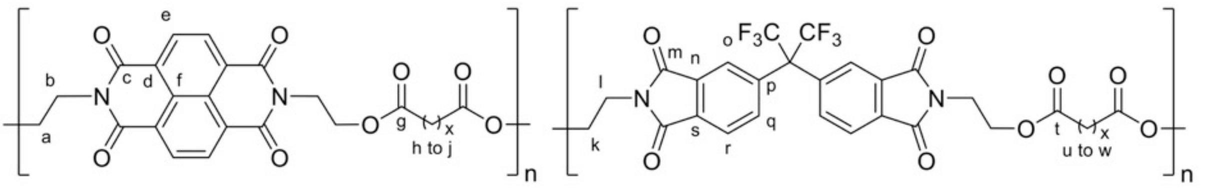

N,N'-Bis-(2-hydroxyethyl)-1,4,5,8-naphthalenetetracarboxylic diimide (1.6051 g, 4.53 mmol mmol), *N,N'*-bis(2-hydroxyethyl)-hexafluoroisopropylidene-diphthalic diimide (2.4450 g, 4.61 mmol), and nonanedioyl dichloride (2.0541 g, 9.12 mmol) were dissolved in anhydrous 1,2-dichlorobenzene (3.0 mL) and the mixture was heated with stirring under a slow nitrogen purge to 120 °C, resulting in evolution of HCl which continued for 30 min. After a total of 60 min. at 130 °C, the viscous solution was cooled to room remperature and taken up in a mixture of dichloromethane and trifluoroethanol (4:1, v/v; 30 mL). The solution was added dropwise with stirring to methanol (400 mL) and the precipitated copolymer was filtered off, washed with methanol, dried under vacuum at 80 °C and finally purified by two further reprecipitations from the same solvent mixture. Yield 4.23 g (75%).

Inherent viscosity (η_{inh}, 25 °C, $CHCl_3$/TFE 6:1, v:v): 1.20 dL g^{-1}. M_n (from viscosity/GPC calibration; p. S6) 44,000. T_g (DSC): 72 °C. FTIR ν_{max} ATR (cm^{-1}): 1780 (imide νC=O), 1706 (ester νC=O), 1387 (imide νC-N), 1189 (vs, νC–F), 1161 (ester νC-O-C). ^1H NMR (400 MHz, $CDCl_3$/TFA 9:1 v:v) δ_H 8.84 (s, $4H_e$), 7.99 (d, *J* = 8.0 Hz, $2H_{q/r}$), 7.94–7.79 (m, $4H_{q/r/o}$), 4.71–4.46 (m, $8H_{a/b}$), 4.39 (t, *J* = 4.9 Hz, $4H_l$), 4.04 (t, *J* = 5.0 Hz, $4H_k$), 2.44–2.23 (m, $8H_{h/u}$), 1.71–1.37 (m, $8H_{i/v}$), 1.37–1.06 (m, $12H_{j/w}$). ^{13}C NMR (101 MHz, $CDCl_3$/TFE 6:1, *v:v*) δ_C 175.21($C_{g/t}$), 175.06 ($C_{g/t}$), 167.57 (C_m), 163.41 (C_c), 139.28 ($C_{n/s}$), 136.27 ($C_{q/r}$), 132.90 (C_p), 132.54 ($C_{n/s}$), 131.53 (C_e), 127.07 ($C_{d/f}$), 126.70 ($C_{d/f}$), 125.16 (C_o), 124.07 ($C_{q/r}$), 61.72 ($C_{a/k}$), 39.80 (C_b), 37.52(C_l) , 34.25 (C_h), 34.18 (C_u), 28.95 ($C_{i/v}$), 24.71 ($C_{j/w}$).

Copolymer 13 (*x* = 8):

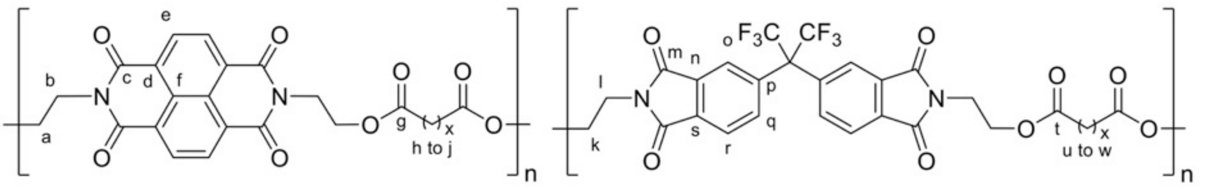

N,N'-Bis-(2-hydroxyethyl)-1,4,5,8-naphthalenetetracarboxylic diimide (1.6103 g, 4.54 mmol mmol), *N,N'*-bis(2-hydroxyethyl)-hexafluoroisopropylidene-diphthalic diimide (2.4645 g, 4.65 mmol), and decanedioyl dichloride (2.2019 g, 9.21 mmol) were dissolved in anhydrous 1,2-dichlorobenzene (3.0 mL) and the mixture was heated with stirring under a slow nitrogen purge to 120 °C, resulting in evolution of HCl which continued for 15 min. After a total of 90 min. at 120 °C, the viscous solution was cooled to room remperature and taken up in a mixture of dichloromethane and trifluoroethanol (4:1, v/v; 20 mL). The solution was added dropwise with stirring to methanol (400 mL) and the precipitated copolymer was filtered off, washed with methanol, dried under vacuum at 80 °C and finally purified by two further reprecipitations from the same solvent mixture. Yield 4.26 g (77%).

Inherent viscosity (η_{inh}, 25 °C, $CHCl_3$/TFE 6:1, v:v): 1.37 dL g^{-1}. M_n (from viscosity/GPC calibration; p. S6) 50,000. T_g (DSC): 65 °C. FTIR ν_{max} ATR (cm^{-1}): 1780 (imide νC=O), 1718 (ester νC=O), 1373 (imide νC-N), 1190 (vs, νC–F), 1162 (ester νC-O-C). ^{1}H NMR (400 MHz, $CDCl_3$/TFA 9:1 v/v) δ 8.84 (s, $4H_e$), 8.11–7.95 (m, $2H_{q/r}$), 7.94–7.79 (m, $4H_{q/r/o}$), 4.70–4.47 (m, $8H_{a/b}$), 4.40 (t, J = 4.9 Hz, $4H_l$), 4.05 (t, J = 4.9 Hz, $4H_k$), 2.56–2.24 (m, $8H_{h/u}$), 1.75–1.42 (m, $8H_{i/v}$), 1.40–1.02 (m, $16H_{j/w}$). ^{13}C NMR (100 MHz, $CDCl_3$/TFE 6:1, *v:v*) δ_C 174.78 $C_{g/t}$, 174.60 ($C_{g/t}$), 167.18 (C_m), 163.05 (C_c), 138.96 ($C_{n/s}$), 135.93 ($C_{q/r}$), 132.66 (C_p), 132.31 ($C_{n/s}$), 131.22 (C_e), 126.77 ($C_{d/f}$), 126.42 ($C_{d/f}$), 124.89 (C_o), 123.78 ($C_{q/r}$), 61.39 ($C_{a/k}$), 39.57 (C_b), 37.30 (C_l), 34.03 (C_h), 33.95(C_u), 28.88 ($C_{i/v}$), 24.51($C_{j/w}$).

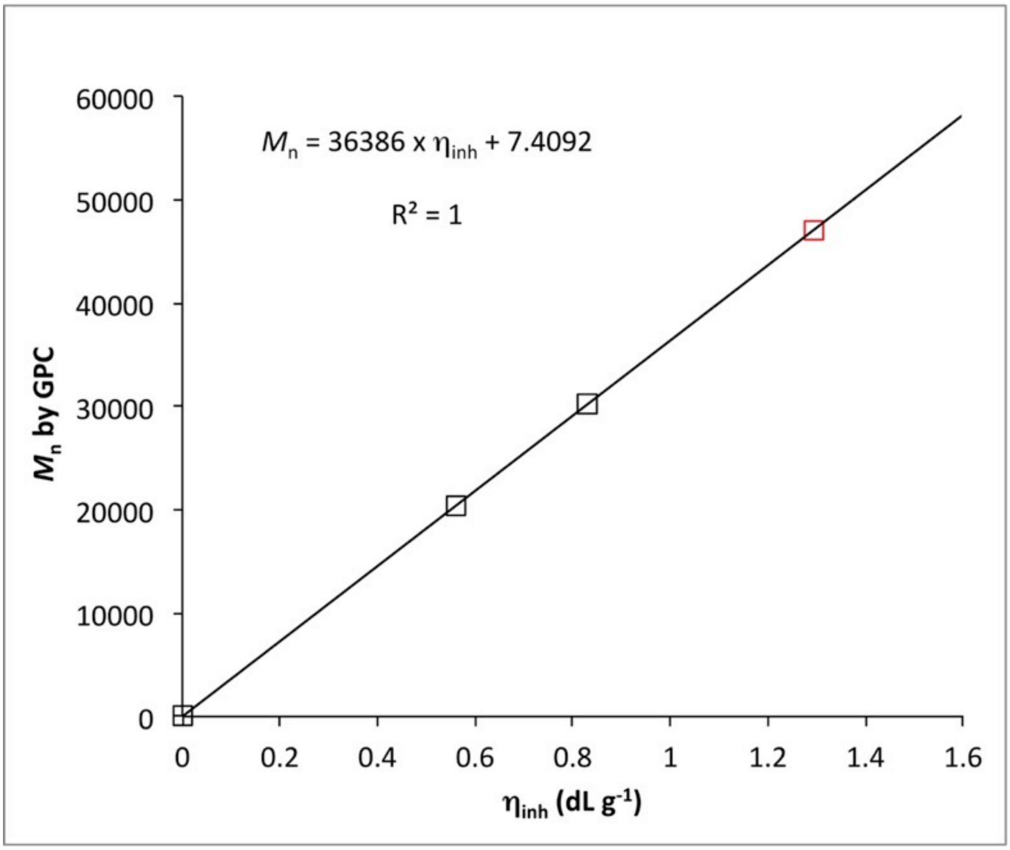

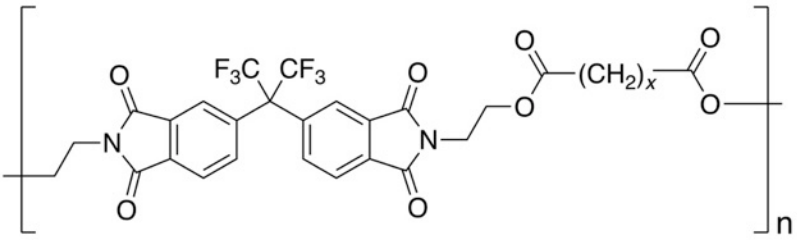

(x = 3, 5)

Figure S1. Calibration plot: Inherent viscosity vs M_n (GPC). The straight-line equation of fit is derived from the zero point and experimental data for the readily-soluble HFDI-based poly(ester-imide)s shown above.[S1] The data point in red, marking the upper range of the present inherent viscosity data, is an extrapolation using the equation of fit for the first three points. This plot was also given in the Supporting Information of a previous publication, (ref. 16 of the present main paper), but is reproduced here for ease of reference.

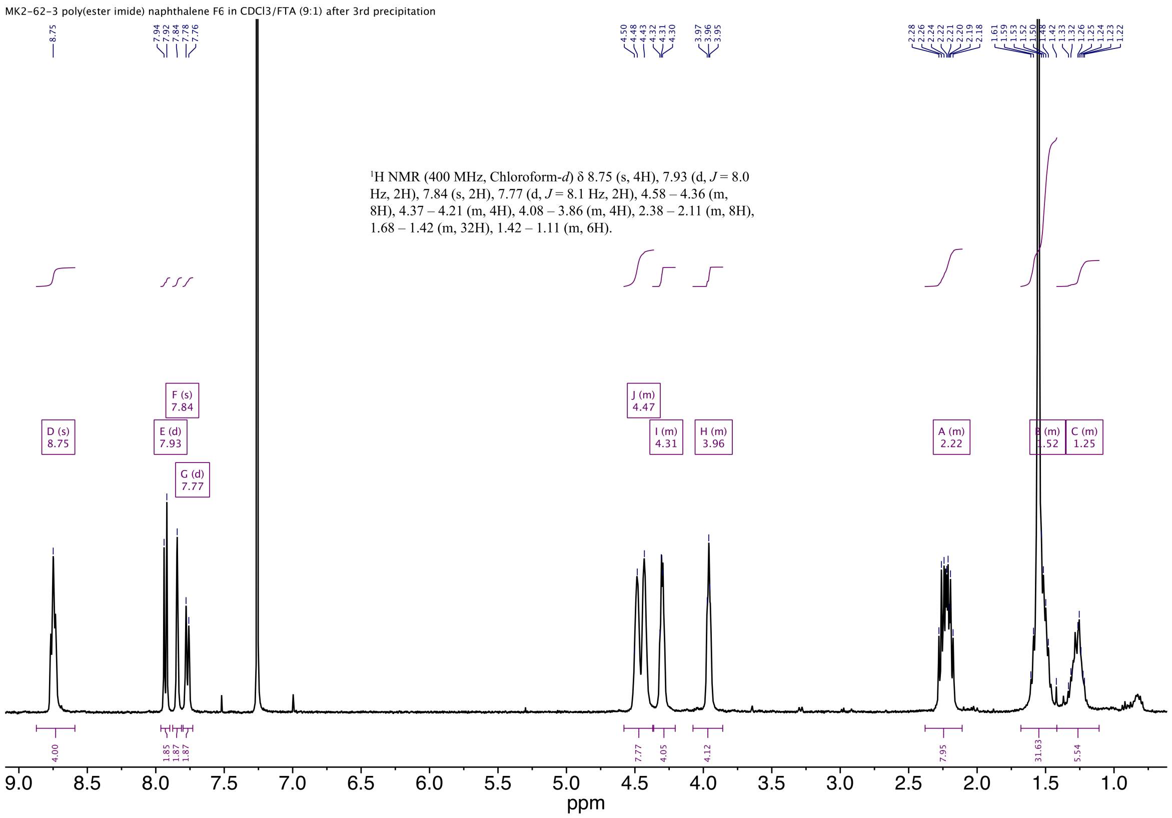

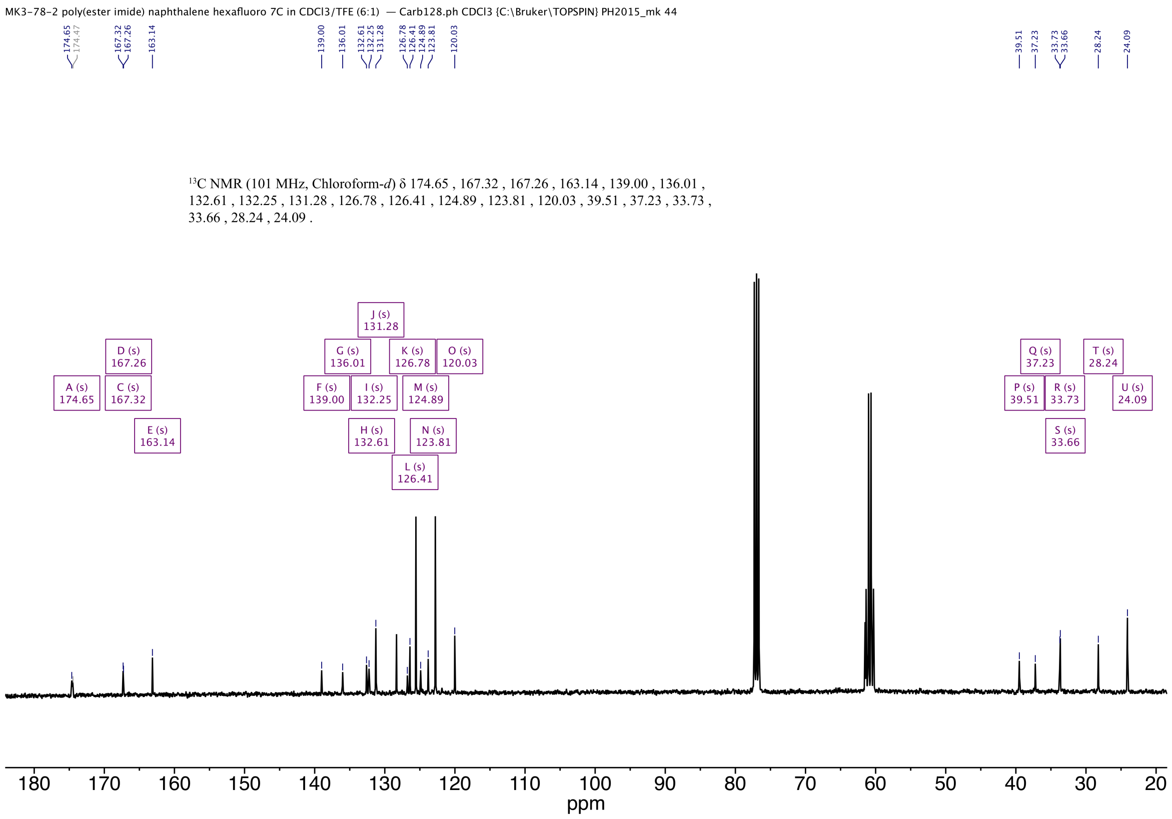

Figure S2. ^{1}H NMR spectrum (above) and ^{13}C NMR spectrum (below) of copolymer **10** (400 MHz).

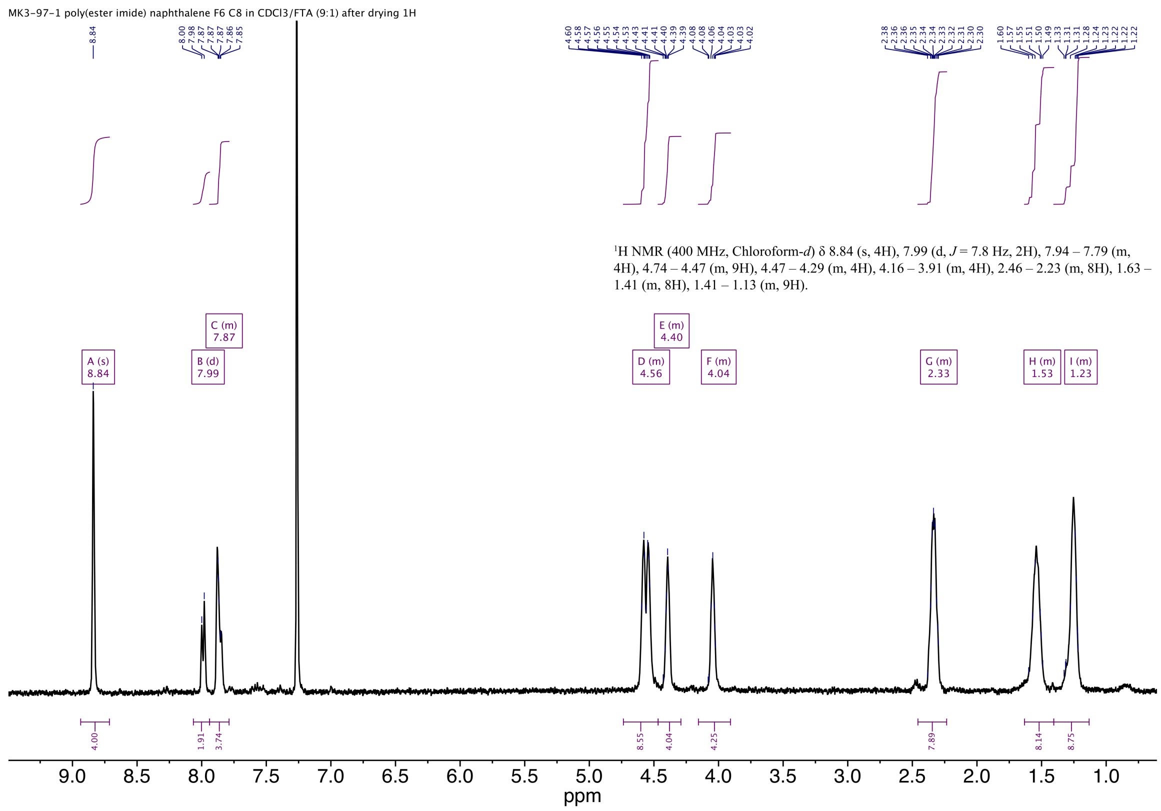

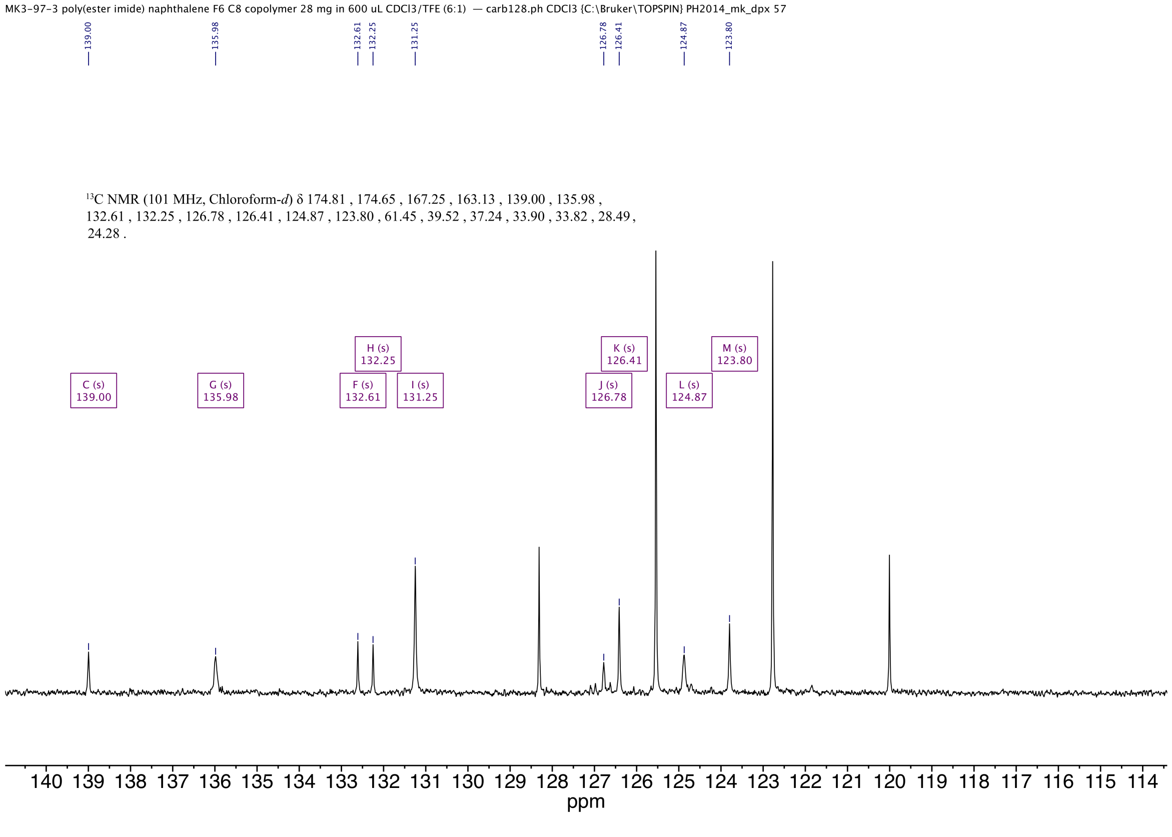

Figure S3. ^{1}H NMR spectrum (above) and ^{13}C NMR spectrum (below) of copolymer **11** (400 MHz).

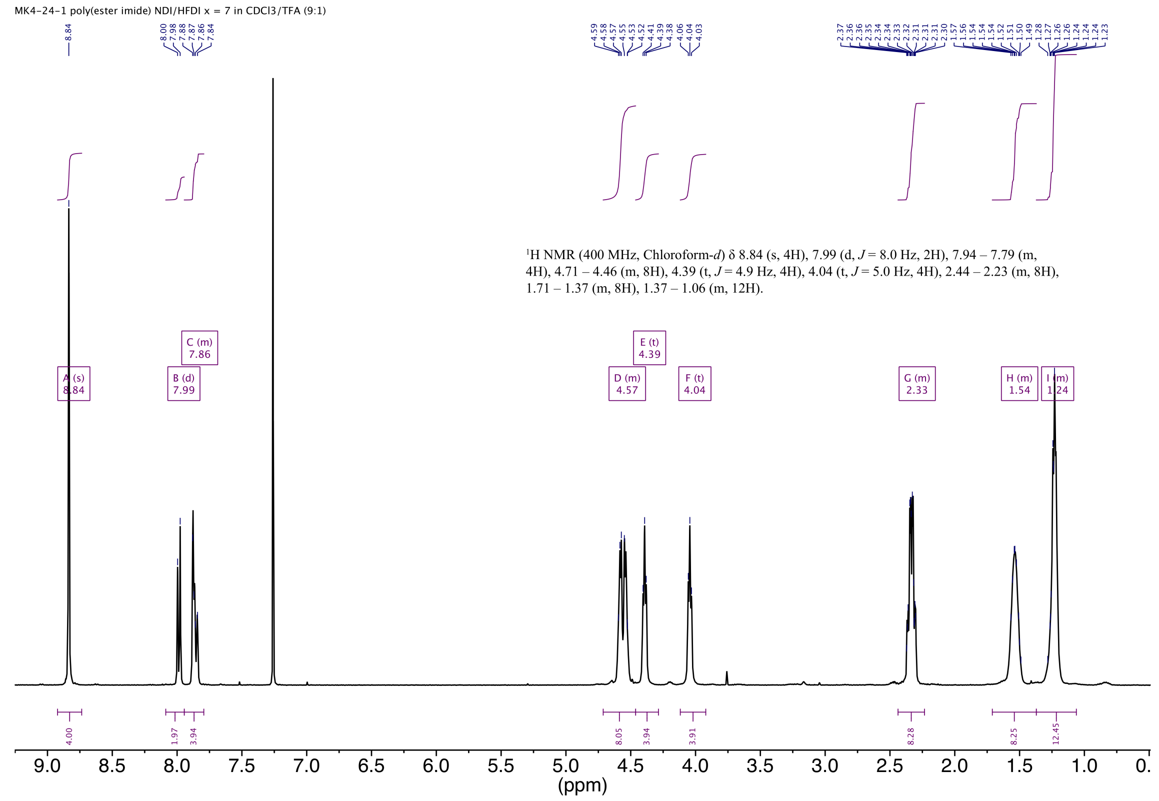

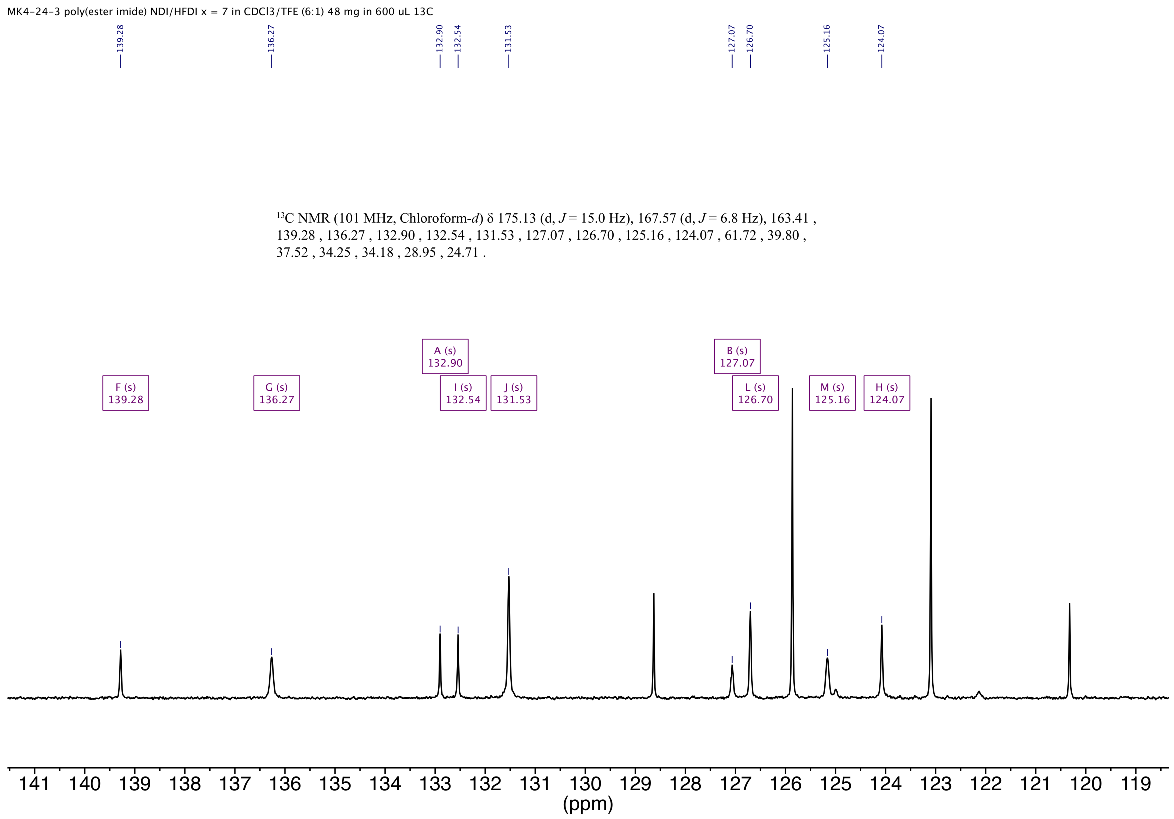

Figure S4. ^{1}H NMR spectrum (above) and ^{13}C NMR spectrum (below) of copolymer **12** (400 MHz)

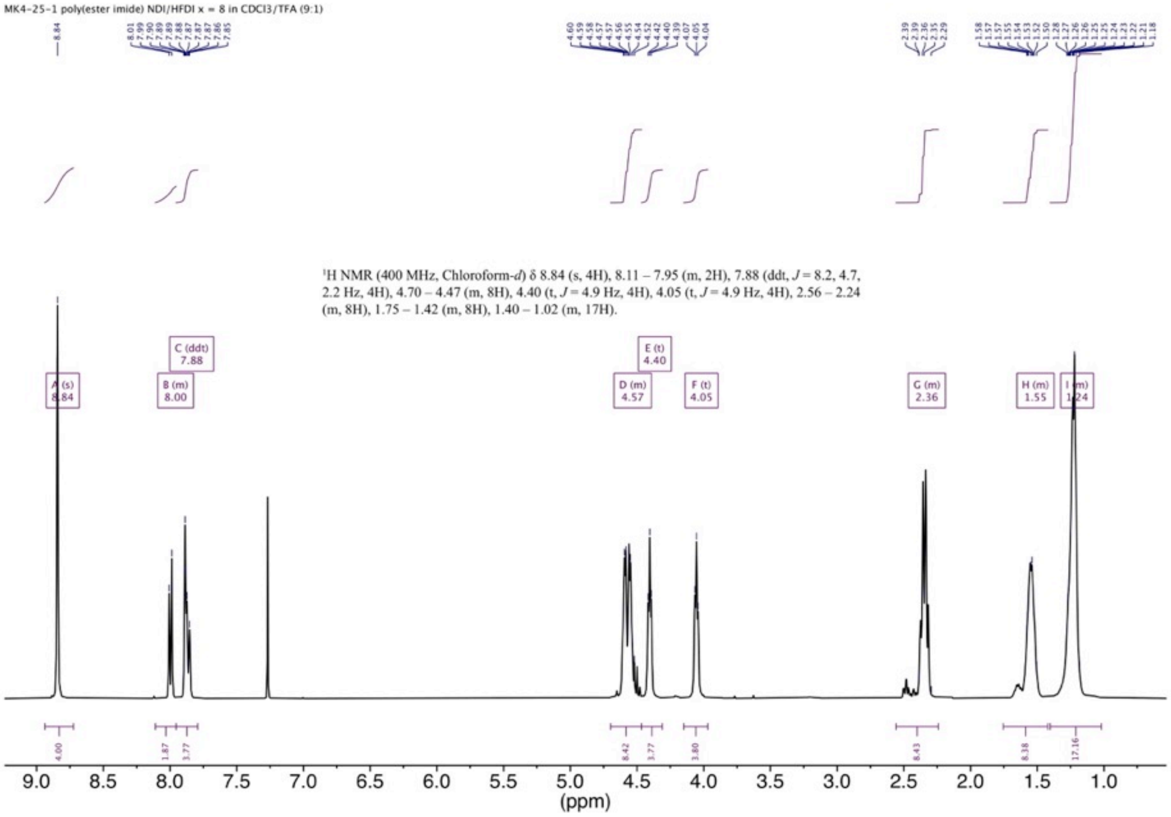

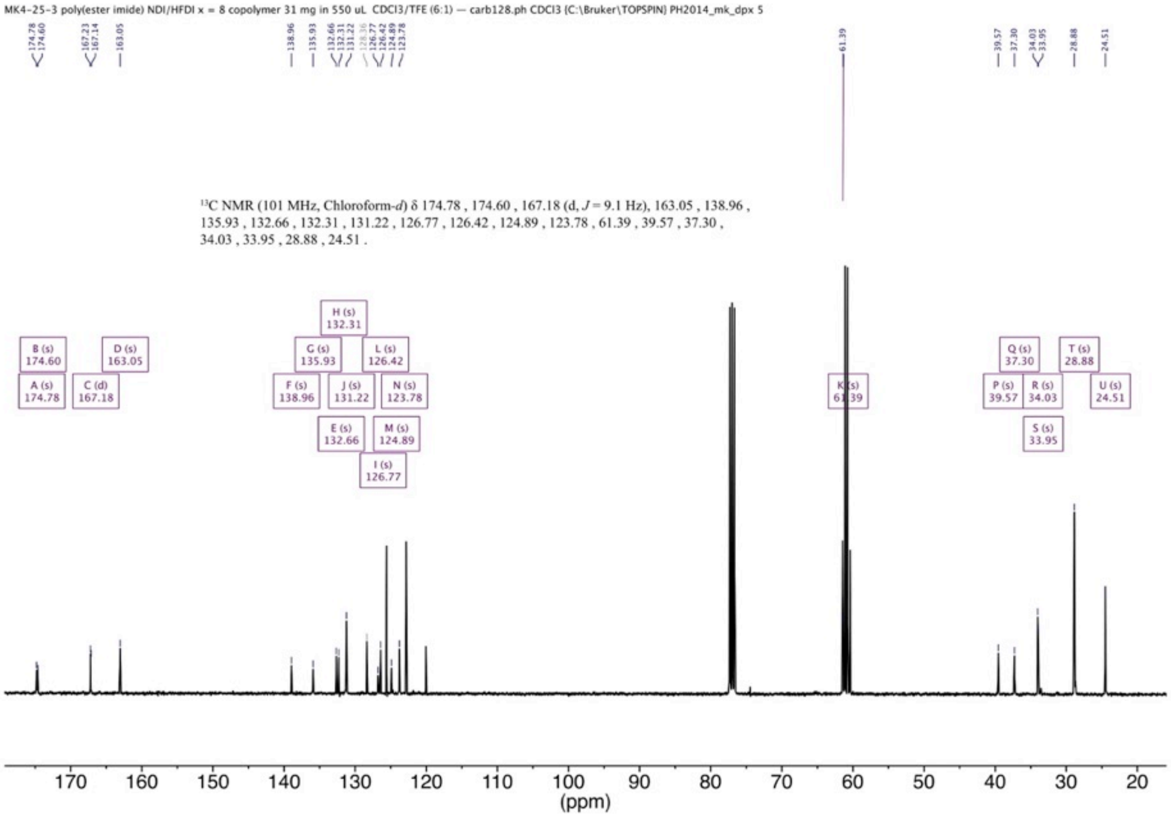

Figure S5. ^{1}H NMR spectrum (above) and ^{13}C NMR spectrum (below) of copolymer **13** (400 MHz)

Protocol for ^{1}H NMR titration at constant concentration of NDI residues

Equimolar stock solutions with exact concentrations of pyrene-d_{10} (48 mM) and a polymer (24 mM with respect to NDI residues) in $CDCl_3$/trifluoroethanol (6 : 1 v/v) were prepared by weighing an approximate amount of pyrene-d_{10} or polymer, respectively, and adding the required volume of solvent via a micropipette to give the desired concentration. Copolymer solution (100 μL) was added to each NMR tube using a micropipette and varying volumes of the pyrene solution were then added to the tubes to give the required molar ratio of pyrene to NDI. The NMR tubes were then filled to 600 μL total volume, by adding the required volume of solvent and were well-mixed, thus affording a constant diimide (NDI) concentration of 4 mM in each tube.

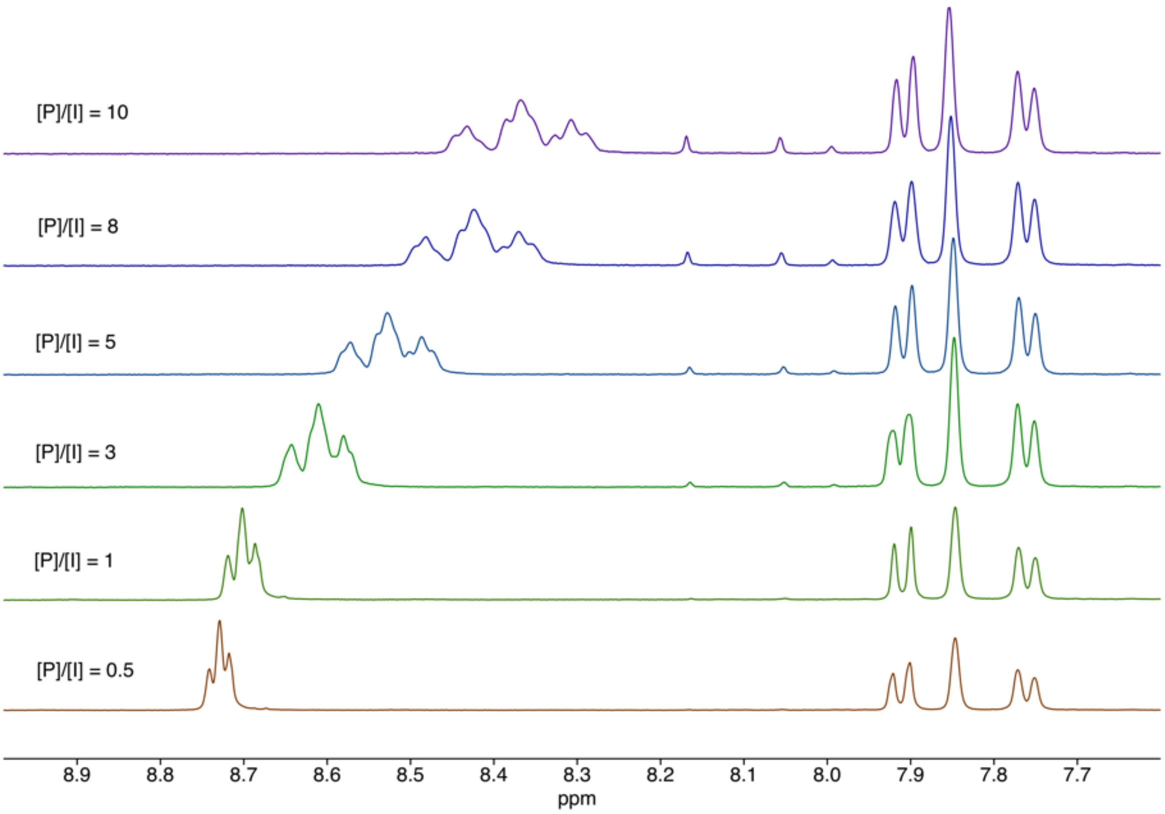

Figure S6. ^{1}H NMR titration (400 MHz, $CDCl_3$/trifluoroethanol, 6:1 v/v) of copolymer **10** (x = 5) against pyrene-d_{10} (0.5 to 10 mole equivalents per NDI residue). Concentration of NDI = 4 mM. [P]/[I] is the molar ratio of pyrene to NDI residues.

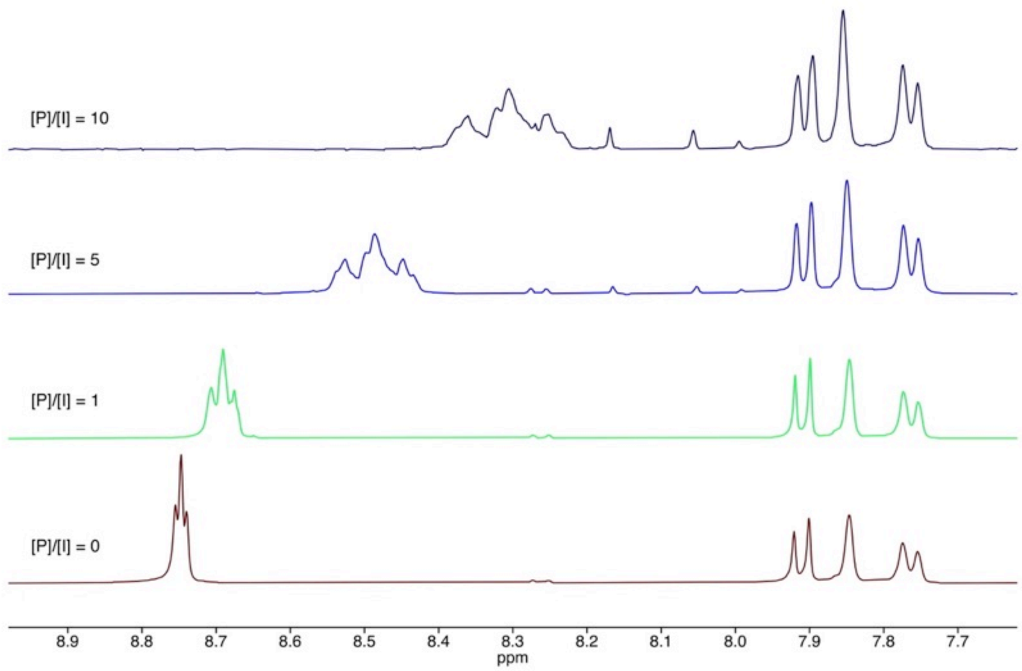

Figure S7. ^{1}H NMR titration (400 MHz, $CDCl_3$/trifluoroethanol, 6:1 v/v) of copolymer **11** (x = 6) against pyrene-d_{10} (0 to 10 mole equivalents per NDI residue). Concentration of NDI = 4 mM. The weak doublet at 8.26 ppm is due to residual 1-chloronaphthalene (polymerisation solvent).

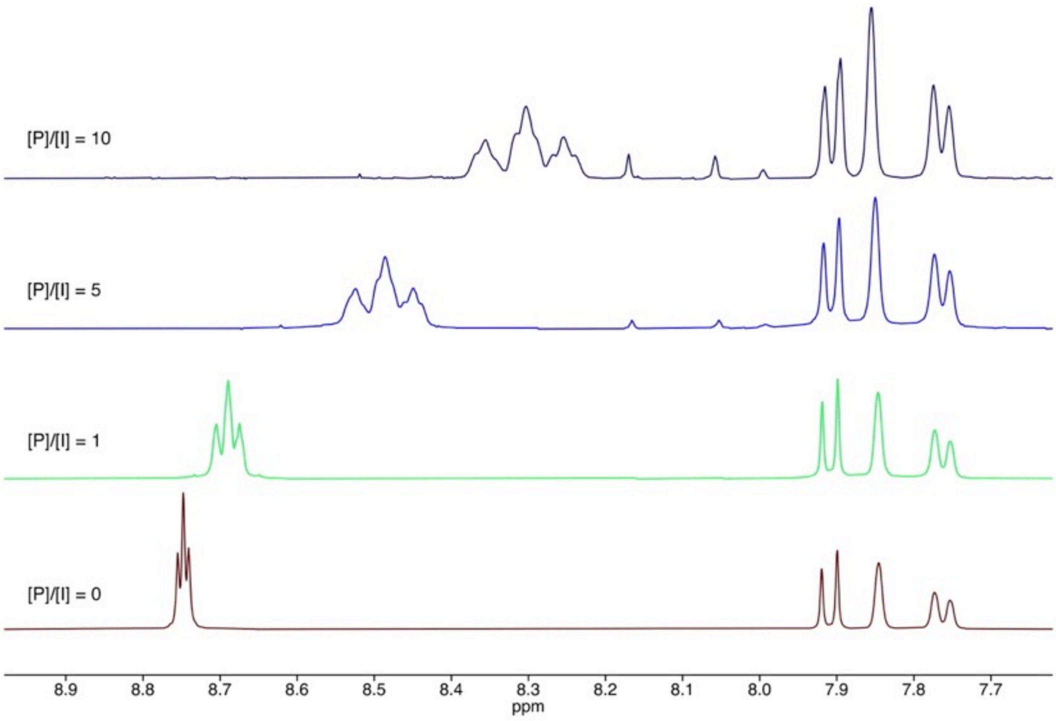

Figure S8. ^{1}H NMR titration (400 MHz, $CDCl_3$/trifluoroethanol, 6:1 v/v) of copolymer **12** (x = 7) against pyrene-d_{10} (0 to 10 mole equivalents per NDI residue). Concentration of NDI = 4 mM. [P]/[I] is the molar ratio of pyrene to NDI residues.

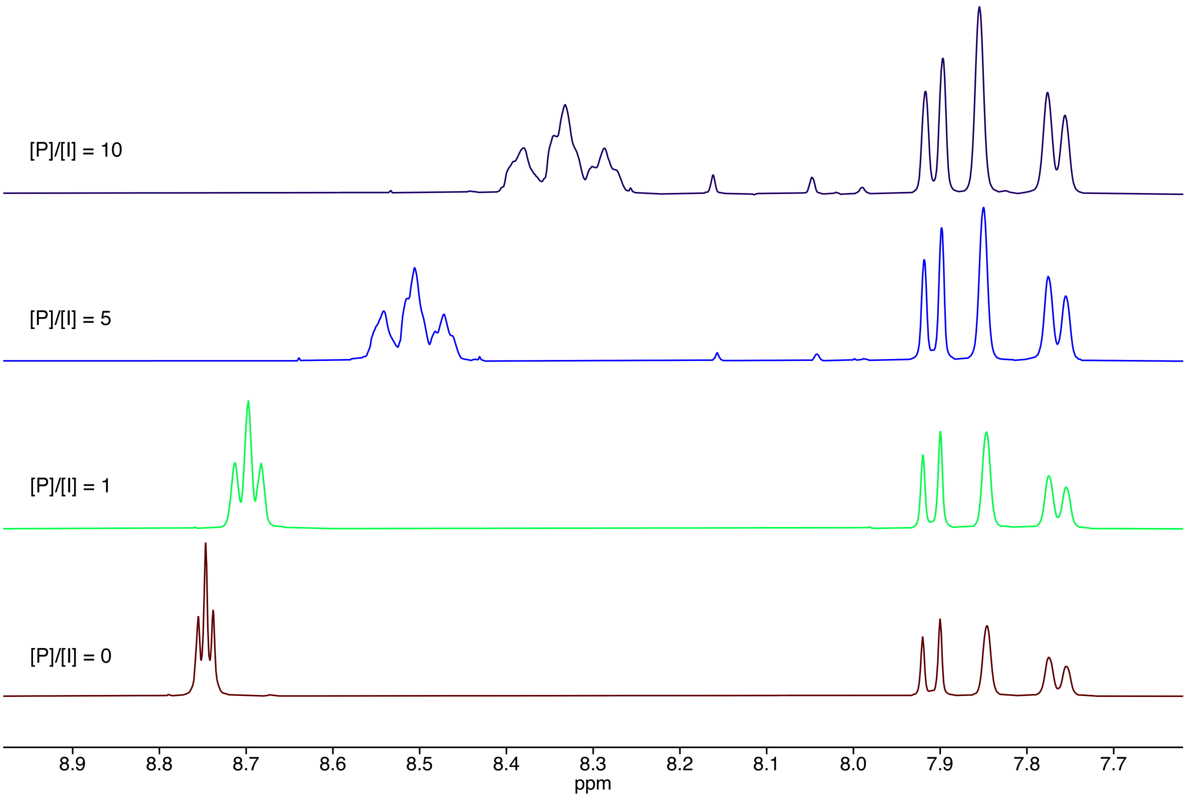

Figure S9. ^{1}H NMR titration (400 MHz, $CDCl_3$/trifluoroethanol, 6:1 v/v) of copolymer **13** (x = 8) against pyrene-d_{10} (0 to 10 mole equivalents per NDI residue). Concentration of NDI = 4 mM. [P]/[I] is the molar ratio of pyrene to NDI residues.

Equation-fitting analysis

In order to obtain the variation of the parameters in the exponential-decay model:

$$T = T_0 + a \sum_{k=1}^{k_{\max}} \frac{N_k}{b^k}$$

with the concentration of pyrene, we use the experimental peaks at molar ratios (of pyrene to NDI residues [P]/[I]) of 3, 5, 8 and 10.

The fitting procedure was as follows. First, at each concentration, we measure the separations between peaks with consecutive values of N_1, with fixed N_2=1. We take the average of the two measurements and call that λ_1.

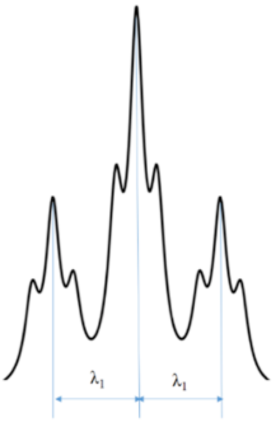

We then measure the separations between peaks with consecutive values of N_2 and the same value of N_1. We take the average of the six equivalent measurements and call that λ_2.

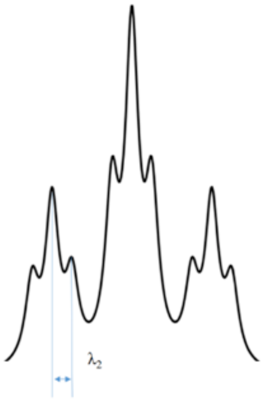

We find the variations of λ_1 and λ_2 with concentration shown in Figure S10:

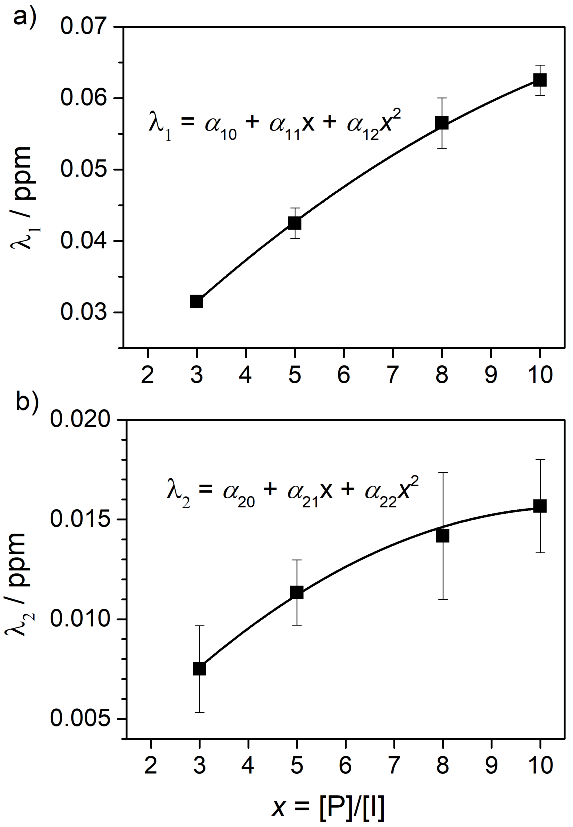

Figure S10. Variation of λ_1 and λ_2 (peak separations of first and second order) with the molar ratio of pyrene to NDI residues. The fitting parameters are $\alpha_{10}=1.05\times10^{-2}$; $\alpha_{11}=7.72\times10^{-3}$; $\alpha_{12}=-2.50\times10^{-4}$; $\alpha_{20}=7.24\times10^{-4}$; $\alpha_{21}=2.65\times10^{-3}$; $\alpha_{22}=-1.17\times10^{-4}$.

The error bars in the plots are obtained from the standard deviations of the measurements described above. There are large error bars for λ_2 as these distances are not much larger than the resolution of the experiment.

Since, due to the resolution of the experiment, we cannot measure peak separations beyond second order, we use $k_{\max} = 2$. In this case:

$$T = T_0 + N_1\lambda_1 + N_2\lambda_2$$

with

$$\lambda_1 = \frac{a}{b}; \quad \lambda_2 = \frac{a}{b^2}$$

then

$$b = \frac{\lambda_1}{\lambda_2}; \quad a = \frac{\lambda_1^2}{\lambda_2}$$

In this way we can univocally determine *a* and *b* for each concentration (the equations above show that no other combination of *a* and *b* values could lead to the experimentally observed peak separations). Finally, T_0 corresponds to the position of the "00" peak, but since that peak is poorly resolved, we calculate T_0 from the position of the well-resolved central "11" peak:

$$T_0 = T(11) - \lambda_1 - \lambda_2.$$

This analysis leads to the results shown in Figure 7 of the main paper. The error bars for all the parameters are propagated based on the equations above.

An alternative quadratic decay model: why it is not satisfactory

As mentioned above, given the resolution of the experiment, we can only measure the peak separations of order one (λ_1) and two (λ_2). We know that these are in a ratio $\frac{\lambda_2}{\lambda_1}$ of approximately ¼ , but from this experiment we cannot establish directly the rate of decay of higher-order contributions, i.e. what the values of $\lambda_3, \lambda_4,$ etc. will be. The model used in this paper, and in the fitting above, assumes an exponential decay such that the higher-order peak separations, if they could be measured, would be represented by $\lambda_3 = \frac{\lambda_1}{16}$; $\lambda_4 = \frac{\lambda_1}{64}$, etc.

It is interesting to observe that a quadratic decay model (in which the ring-current shielding decays as the square of the distance from the binding NDI residue to the central, "observed" NDI) is also consistent with the measured $\frac{\lambda_2}{\lambda_1}$ ratio. In this case, the model could be written as:

$$T = T_0 + \alpha \sum_{k=1}^{k_{\max}} \frac{N_k}{(kd)^2}$$

where *kd* is distance from the binding site to the centre. Because the distance doubles from *k*=1 to *k*=2, the peak separations of order one and two are:

$$\lambda_1 = \frac{\alpha}{d^2}; \quad \lambda_2 = \frac{\alpha}{(2d)^2}$$

which are again in the observed ratio $\frac{\lambda_2}{\lambda_1}$ = ¼. However, we show here that a quadratic decay is not consistent with the current experiment, nor with data reported in Ref. 15 (main paper).

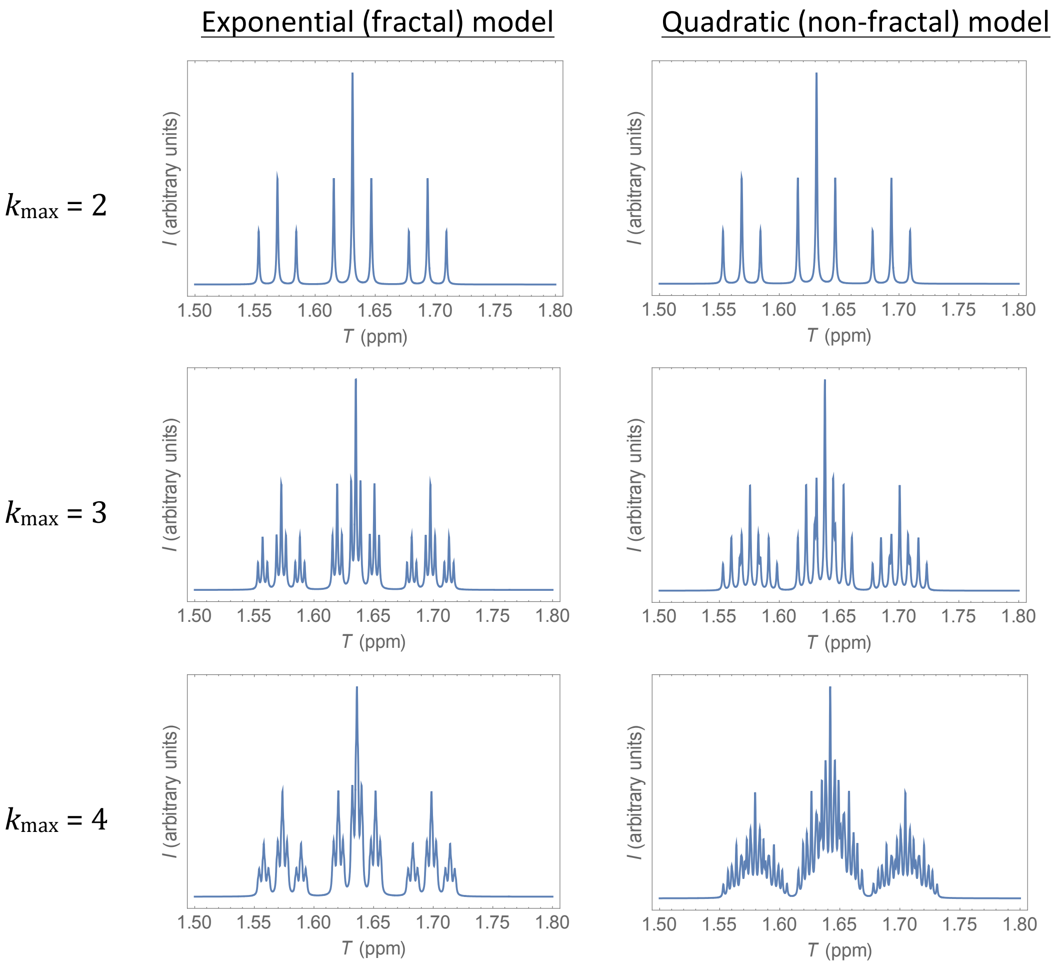

Figure S11. Simulated NMR spectra for [P]/[I] = 10 using either exponential decay (fractal model) or quadratic decay (non-fractal model). A linewidth of 0.5 Hz is used in these simulations to resolve the individual peaks.

The problem with the quadratic model is that the peaks of higher order are too widely separated, so their inclusion in the simulation leads to results that are incompatible with experiment. This is illustrated in Figures S11 and S12, where we extend the simulation to higher orders of peaks, to model the best-resolved spectrum, which is the one with [P]/[I]=10 (λ_1=00625 ppm; λ_2=0.0157 ppm).

Figure S11 shows the simulations with an artificially narrow linewidth of 0.5 Hz, to resolve the individual peaks. Whereas for k_{max} = 2 (quintets) the predictions from the exponential and quadratic models are identical, when higher-order peaks are included in the simulations the behaviours of the two models differ very considerably. The higher-order peaks from the quadratic model are much more widely separated ($\lambda_3 = \frac{\lambda_1}{9}$; $\lambda_4 = \frac{\lambda_1}{16}$) than in the exponential

model $(\lambda_3 = \frac{\lambda_1}{16};\ \lambda_4 = \frac{\lambda_1}{64})$. As a result, in the quadratic model, the peaks fill all the gaps between resonances, contrasting with the retention of gaps (the fractal "tremas" – to use a term coined by Mandelbrot) in the exponential model. Thus, when the spectrum is simulated at a more realistic linewidth of 4 Hz (Figure S12), inclusion of higher-order terms (up to k_{max} = 4) in the quadratic model leads to the "loss" of the second order peaks, which are perfectly visible in experiment. In contrast, increasing the expansion cutoff, k_{max}, in the exponential model leads to a converged result that is in good agreement with the experimental spectrum.

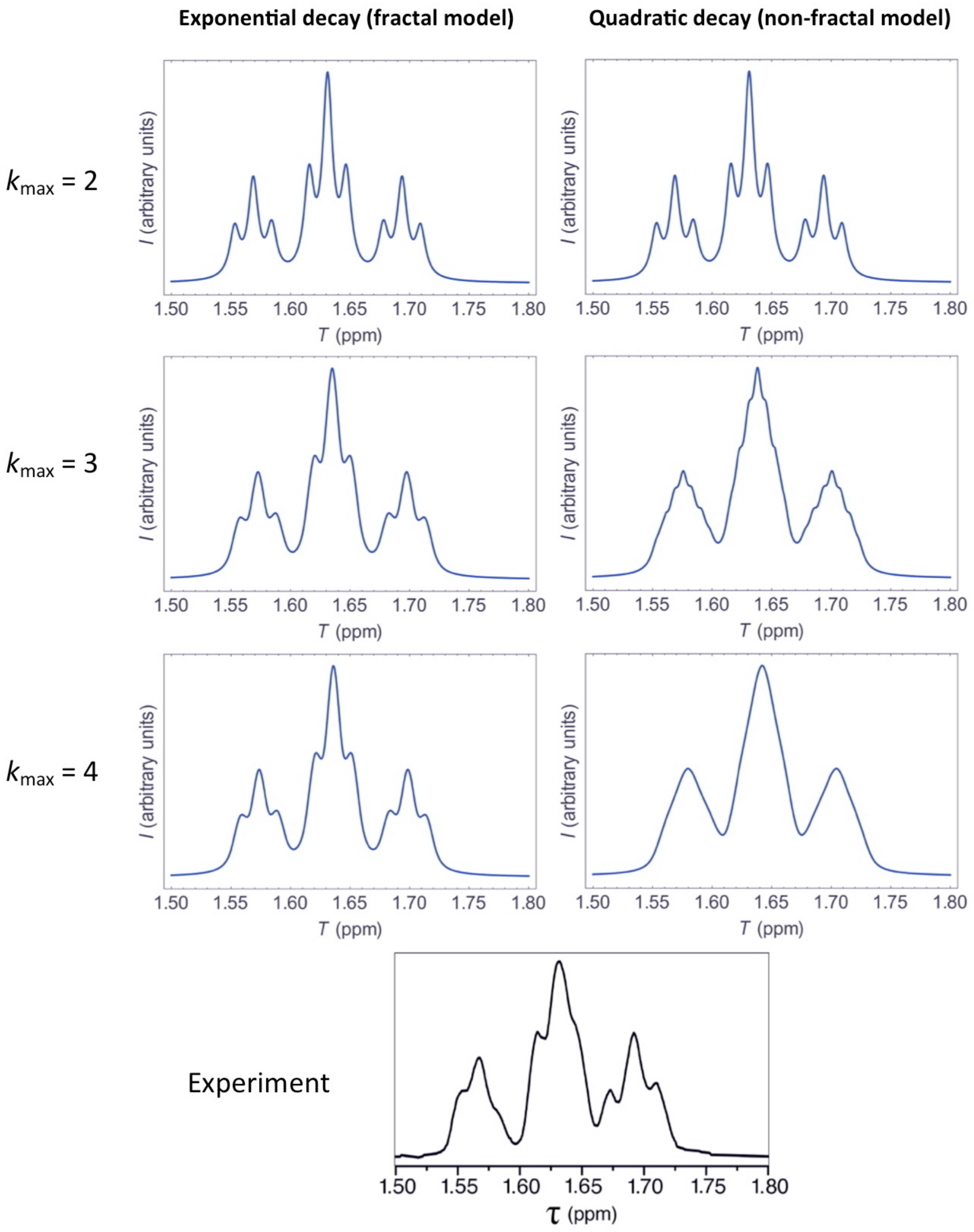

Figure S12. Simulated NMR spectra for [P]/[I] = 10 using either exponential decay (fractal model) or quadratic decay (non-fractal model), in comparison with the experimental spectrum for [P]/[I] = 10. A linewidth of 4 Hz is used in the simulated spectra.

We have also re-visited the data in our previous work (Ref. 15, main paper) to test whether a quadratic model could have described it. In that work, we were able to resolve peak separations of up to third order, so here we do not need to rely on broadening-analysis to compare the exponential and quadratic models. The comparison of the two models with experiment are shown in Figure S13 a and b. Clearly, the third-order peak separations are much better described by an exponential model than by a quadratic one. The reason is the same as that discussed above: a quadratic model overestimates higher-order peak separations.

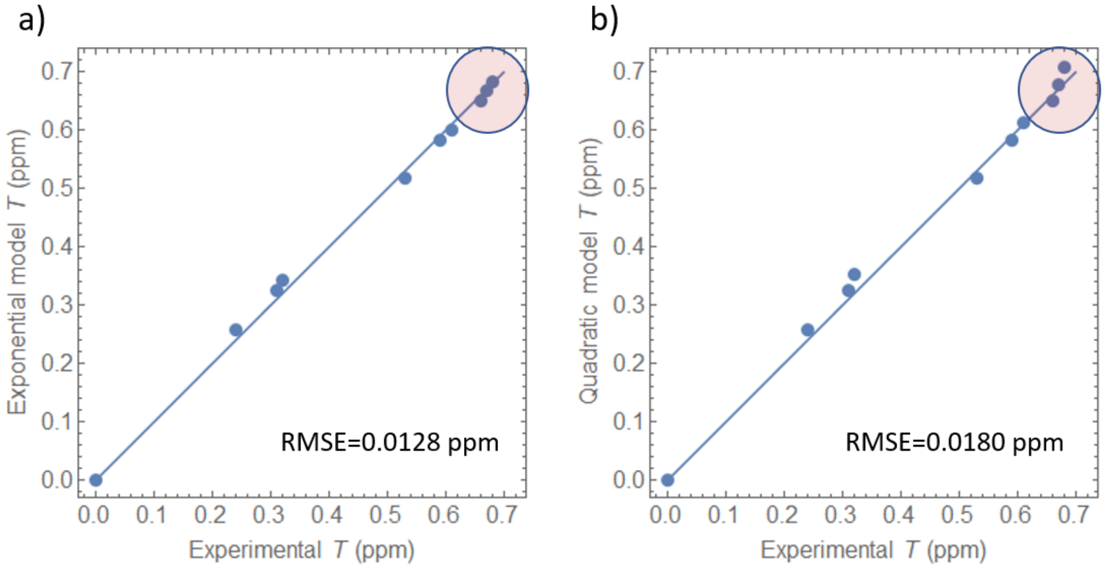

Figure S13. Comparison of predictions from a) exponential model and b) quadratic model with the experimental data reported in Ref. 15 (main paper). The shaded circles enclose points separated by third-order contributions. The straight line represents the $y = x$ ideal match. The RMSE values are root-mean-square errors.

Methodology and calculation tables for ^{1}H NMR simulations

Simulations of ^{1}H NMR spectra for copolymer **10** in the presence of pyrene (0.5 to 10 equivalents per NDI residue) were carried out using the "Peak Table to Spectrum" script within Mestrenova, version 14.1. Input data (chemical shifts) were obtained from Equation **3** using parameters *a*, *b* (= 4) and T_0 derived from the fitting protocol described on p.S15. Relative intensities were equated to degeneracies, Ω, as derived from Equation **2**. Thus:

a = 0.0175[P]/[I] + 0.0776

b = 4

T_0 = 0.029[P]/[I] + 1.268

$$T = T_0 + a \sum_{k=1}^{2} \frac{N_k}{b^k}$$

Equation **3**

$$\frac{I}{I_{\max}} = \frac{\Omega}{\Omega_{\max}} = \frac{1}{\prod_k 2^{1-\delta_{1,N_k}}}$$

Equation **2**

==================

Quintet sequences, two-digit codes, second iteration, nine resonances

For [P]/[I] = **0.5**

a = 0.0864

T_0 = 1.2825

N_k	T_0	*T* Calculation	*T* (tau)	*T* (delta)	Degen.
00	1.2825	1.2825 + 0.0864(0+0)	1.2825	8.7175	1
01	1.2825	1.2825 + 0.0864(0 + 1/16)	1.2879	8.7121	2
02	1.2825	1.2825 + 0.0864(0 + 2/16)	1.2933	8.7067	1
10	1.2825	1.2825 + 0.0864(1/4 +0)	1.3041	8.6959	2
11	1.2825	1.2825 + 0.0864(1/4 + 1/16)	1.3095	8.6905	4
12	1.2825	1.2825 + 0.0864(1/4 + 2/16)	1.3149	8.6851	2
20	1.2825	1.2825 + 0.0864(2/4 + 0)	1.3257	8.6743	1
21	1.2825	1.2825 + 0.0864(2/4 + 1/16)	1.3311	8.6689	2
22	1.2825	1.2825 + 0.0864(2/4 + 2/16)	1.3365	8.6635	1

For [P]/[I] = **3**

a = 0.1301
T_0 = 1.3550

N_k	T_0	T Calculation	T (tau)	T (delta)	Degen.
00	1.3550	1.3550 + 0.1301(0+0)	1.3550	8.6450	1
01	1.3550	1.3550 + 0.1301(0 + 1/16)	1.3631	8.6369	2
02	1.3550	1.3550 + 0.1301(0 + 2/16)	1.3713	8.6287	1
10	1.3550	1.3550 + 0.1301(1/4 +0)	1.3875	8.6125	2
11	1.3550	1.3550 + 0.1301(1/4 + 1/16)	1.3957	8.6043	4
12	1.3550	1.3550 + 0.1301(1/4 + 2/16)	1.4039	8.5961	2
20	1.3550	1.3550 + 0.1301(2/4 + 0)	1.4201	8.5799	1
21	1.3550	1.3550 + 0.1301(2/4 + 1/16)	1.4282	8.5718	2
22	1.3550	1.3550 + 0.1301(2/4 + 2/16)	1.4363	8.5637	1

For [P]/[I] = **5**

a = 0.1651
T_0 = 1.4130

N_k	T_0	T Calculation	T (tau)	T (delta)	Degen.
00	1.4130	1.4130 + 0.1651(0+0)	1.4130	8.5870	1
01	1.4130	1.4130 + 0.1651(0 + 1/16)	1.4233	8.5767	2
02	1.4130	1.4130 + 0.1651(0 + 2/16)	1.4336	8.5664	1
10	1.4130	1.4130 + 0.1651(1/4 +0)	1.4543	8.5457	2
11	1.4130	1.4130 + 0.1651(1/4 + 1/16)	1.4646	8.5354	4
12	1.4130	1.4130 + 0.1651(1/4 + 2/16)	1.4749	8.5251	2
20	1.4130	1.4130 + 0.1651(2/4 + 0)	1.4956	8.5044	1
21	1.4130	1.4130 + 0.1651(2/4 + 1/16)	1.5059	8.4941	2
22	1.4130	1.4130 + 0.1651(2/4 + 2/16)	1.5162	8.4838	1

For [P]/[I] = **8**

a = 0.2176
T_0 = 1.5000

N_k	T_0	T Calculation	T (tau)	T (delta)	Degen.
00	1.5000	1.5000 + 0.2176(0+0)	1.5000	8.5000	1
01	1.5000	1.5000 +0.2176(0 + 1/16)	1.5136	8.4864	2
02	1.5000	1.5000 + 0.2176(0 + 2/16)	1.5272	8.4728	1
10	1.5000	1.5000 + 0.2176(1/4 +0)	1.5544	8.4456	2
11	1.5000	1.5000 + 0.2176(1/4 + 1/16)	1.5680	8.4320	4
12	1.5000	1.5000 + 0.2176(1/4 + 2/16)	1.5816	8.4184	2
20	1.5000	1.5000 + 0.2176(2/4 + 0)	1.6088	8.3912	1
21	1.5000	1.5000 + 0.2176 (2/4 + 1/16)	1.6224	8.3776	2
22	1.5000	1.5000 + 0.2176(2/4 + 2/16)	1.6360	8.3640	1

For [P]/[I] = **10**

a = 0.2526
T_0 = 1.5580

N_k	T_0	***T*** Calculation	T (tau)	T (delta)	Degen.
00	1.5580	1.5580 + 0.2526(0+0)	1.5580	8.4420	1
01	1.5580	1.5580 +0.2526(0 + 1/16)	1.5738	8.4262	2
02	1.5580	1.5580 + 0.2526(0 + 2/16)	1.5896	8.4104	1
10	1.5580	1.5580 + 0.2526(1/4 +0)	1.6212	8.3788	2
11	1.5580	1.5580 + 0.2526(1/4 + 1/16)	1.6369	8.3631	4
12	1.5580	1.5580 + 0.2526(1/4 + 2/16)	1.6527	8.3473	2
20	1.5580	1.5580 + 0.2526(2/4 + 0)	1.6843	8.3157	1
21	1.5580	1.5580 + 0.2526 (2/4 + 1/16)	1.7001	8.2999	2
22	1.5580	1.5580 + 0.2526(2/4 + 2/16)	1.7159	8.2841	1

Septet sequences, three-digit codes, third iteration, twenty-seven resonances

For [P]/[I] = **10**

$$T = T_0 + a \sum_{k=1}^{3} \frac{N_k}{4^k}$$

a = 0.2526
T_0 = 1.5580

N_k	T_0	*T* Calculation	*T* (tau)	*T* (delta)	Degen.
000	1.5580	1.5580 + 0.2526(0 + 0 + 0)	1.5580	8.4420	1
001	1.5580	1.5580 +0.2526(0 + 0 + 1/64)	1.5619	8.4381	2
002	1.5580	1.5580 + 0.2526(0 + 0 + 2/64)	1.5659	8.4341	1
010	1.5580	1.5580 + 0.2526(0 + 1/16 +0)	1.5738	8.4262	2
011	1.5580	1.5580 + 0.2526(0 + 1/16 + 1/64)	1.5777	8.4223	4
012	1.5580	1.5580 + 0.2526(0 + 1/16 + 2/64)	1.5817	8.4183	2
020	1.5580	1.5580 + 0.2526(0 + 2/16 + 0)	1.5896	8.4104	1
021	1.5580	1.5580 + 0.2526(0 + 2/16 + 1/64)	1.5935	8.4065	2
022	1.5580	1.5580 + 0.2526(0 + 2/16 + 2/64)	1.5975	8.4025	1
100	1.5580	1.5580 + 0.2526(1/4 + 0 + 0)	1.6212	8.3788	2
101	1.5580	1.5580 +0.2526(1/4 + 0 + 1/64)	1.6251	8.3749	4
102	1.5580	1.5580 + 0.2526(1/4 + 0 + 2/64)	1.6290	8.3710	2
110	1.5580	1.5580 + 0.2526(1/4 + 1/16 +0)	1.6369	8.3631	4
111	1.5580	1.5580 + 0.2526(1/4 + 1/16 + 1/64)	1.6409	8.3591	8
112	1.5580	1.5580 + 0.2526(1/4 + 1/16 + 2/64)	1.6448	8.3552	4
120	1.5580	1.5580 + 0.2526(1/4 + 2/16 + 0)	1.6527	8.3473	2
121	1.5580	1.5580 + 0.2526(1/4 + 2/16 + 1/64)	1.6567	8.3433	4
122	1.5580	1.5580 + 0.2526(1/4 + 2/16 + 2/64)	1.6606	8.3394	2
200	1.5580	1.5580 + 0.2526(2/4 + 0 + 0)	1.6843	8.3157	1
201	1.5580	1.5580 +0.2526(2/4 + 0 + 1/64)	1.6882	8.3118	2
202	1.5580	1.5580 + 0.2526(2/4 + 0 + 2/64)	1.6922	8.3078	1
210	1.5580	1.5580 + 0.2526(2/4 + 1/16 +0)	1.7001	8.2999	2
211	1.5580	1.5580 + 0.2526(2/4 + 1/16 + 1/64)	1.7040	8.2960	4
212	1.5580	1.5580 + 0.2526(2/4 + 1/16 + 2/64)	1.7080	8.2920	2
220	1.5580	1.5580 + 0.2526(2/4 + 2/16 + 0)	1.7159	8.2841	1
221	1.5580	1.5580 + 0.2526(2/4 + 2/16 + 1/64)	1.7198	8.2802	2
222	1.5580	1.5580 + 0.2526(2/4 + 2/16 + 2/64)	1.7238	8.2762	1
